# Past, present, and future geographic range of the relict Mediterranean and Macaronesian *Juniperus phoenicea* complex

**DOI:** 10.1002/ece3.7395

**Published:** 2021-03-25

**Authors:** Montserrat Salvà‐Catarineu, Angel Romo, Małgorzata Mazur, Monika Zielińska, Pietro Minissale, Ali A. Dönmez, Krystyna Boratyńska, Adam Boratyński

**Affiliations:** ^1^ Department of Geography Universitat de Barcelona Barcelona Spain; ^2^ Botanical Institute of Spanish National Research Council CSIC Barcelona Spain; ^3^ Department of Botany Kazimierz Wielki University Bydgoszcz Poland; ^4^ Department of Biological, Geological and Environmental Sciences University of Catania Catania Italy; ^5^ Faculty of Science Department of Botany Hacettepe University Ankara Turkey; ^6^ Institute of Dendrology Polish Academy of Sciences Kórnik Poland

**Keywords:** biodiversity, biogeography, climate change, *Juniperus canariensis*, *Juniperus phoenicea*, *Juniperus turbinata*, niche modeling, relict tree

## Abstract

**Aim:**

The aim of this study is to model the past, current, and future distribution of *J. phoenicea s.s*., *J. turbinata*, and *J. canariensis*, based on bioclimatic variables using a maximum entropy model (Maxent) in the Mediterranean and Macaronesian regions.

**Location:**

Mediterranean and Macaronesian.

**Taxon:**

Cupressaceae, Juniperus.

**Methods:**

Data on the occurrence of the *J. phoenicea* complex were obtained from the Global Biodiversity Information Facility (GBIF.org), the literature, herbaria, and the authors’ field notes. Bioclimatic variables were obtained from the WorldClim database and Paleoclim. The climate data related to species localities were used for predictions of niches by implementation of Maxent, and the model was evaluated with ENMeval.

**Results:**

The potential niches of *Juniperus phoenicea* during the Last Interglacial period (LIG), Last Glacial Maximum climate (LGM), and Mid‐Holocene (MH) covered 30%, 10%, and almost 100%, respectively, of the current potential niche. Climate warming may reduce potential niches by 30% in RCP2.6 and by 90% in RCP8.5. The potential niches of *Juniperus turbinata* had a broad circum‐Mediterranean and Canarian distribution during the LIG and the MH; its distribution extended during the LGM when it was found in more areas than at present. The predicted warming in scenarios RCP2.6 and RCP8.5 could reduce the current potential niche by 30% and 50%, respectively. The model did not find suitable niches for *J. canariensis* during the LIG and the LGM, but during the MH its potential niche was 30% larger than at present. The climate warming scenario RCP2.6 indicates a reduction in the potential niche by 30%, while RCP8.5 so indicates a reduction of almost 60%.

**Main conclusions:**

This research can provide information for increasing the protection of the juniper forest and for counteracting the phenomenon of local extinctions caused by anthropic pressure and climate changes.

## Graphical abstract

The aim of this study is to model the past, current, and future distribution of *J. phoenicea s.s*., *J. turbinata*, and *J. canariensis*, based on bioclimatic variables using a maximum entropy model (MaxEnt) in the Mediterranean and Macaronesian regions. Climate warming could reduce potential niches of Juniperus phoenicea by 30% and 90% in scenarios RCP2.6 and RCP8.5, respectively.

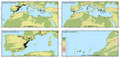

## 1 INTRODUCTION

The geographical distribution of a species is constrained by abiotic variables, predominantly climatic (Guisan & Zimmermann, 2000; Pulliam, 2000; Walas et al., 2019), by biotic interactions (Sexton et al., 2009; Wisz et al., 2013), and by seed dispersal ability (Soberón & Peterson, 2005; Svenning & Skov, 2007). In the Mediterranean Basin, paleogeography and geographical isolation also play an important role, especially for species with low dispersal ability (Hardion et al., 2016; Mansion et al., 2008; Sciandrello et al., 2015).

Ecological Niche Models (ENMs) are predictive tools that assume that species distribution is determined by climate conditions (Di Pasquale et al., 2020; Franklin et al., 2009; Li et al., 2020; Rodríguez‐Sánchez & Arroyo, 2008; Walas et al., 2019) and use their current geographic ranges and climate conditions to predict past and future distribution. The use of georeferenced species localities together with bioclimatic data allows the retro‐ and prospective analysis of their potential niches (Phillips et al., 2006, 2017). Retrospective and prospective niche modeling has been used only occasionally for circum‐Mediterranean and Macaronesian species (Di Pasquale et al., 2020; Rodríguez‐Sánchez & Arroyo, 2008), and also for species occurring within the region (Arar et al., 2020; Benítez‐Benítez et al., 2018; Hajar et al., 2010; Stephan et al., 2020; Taib et al., 2020; Walas et al., 2019). Mediterranean and Macaronesian regions are important biodiversity hotspots at the global scale. However, they have been greatly modified by human activity for millennia and they are vulnerable to current and future climate change (Otto et al., 2012; Thompson, 2005).

The Phoenician juniper is a species complex native to the Mediterranean and Macaronesian regions (as defined by Takhtajan, 1986), where it is a relict from the Tertiary period (Mao et al., 2010). Phylogenetically, it is an old species that developed from an ancestral taxon from the Section *Sabina* Spach, genus *Juniperus* L. (Mao et al., 2010, 2019). The *J. phoenicea* complex includes three species: *J. phoenicea* L. *sensu* *stricto* (*s.s*.), *J. turbinata* Guss., and *J. canariensis* Guyot in Mathou & Guyot (Romo et al., 2019). The species differ from each other in terms of genetics (Adams, 2014; Adams et al., 2002, 2009, 2010, 2013, 2014; Boratyński et al., 2009; Dzialuk et al., 2011; Jiménez et al., 2017; Sánchez‐Gómez et al., 2018), biochemistry (Adams et al., 2002, 2009; Lebreton & Pérez de Paz, 2001; Lebreton & Rivera, 1989; Lebreton & Thivend, 1981), morphological characters of cones and seeds (Mazur et al., 2010, 2016, 2018; Pinna et al., 2014), and phenology (Romo et al., 2019). All three species of *J. phoenicea aggr*. are small trees or large shrubs. Like most junipers, they are light demanding, moderately thermophilic and relatively drought‐resistant (Asensi et al., 2007; Browicz & Zieliński, 1982; Charco, 1999; Lloret & García, 2016; Otto et al., 2012; Quézel & Médail, 2003; Rubio‐Casal et al., 2010; Zohary, 1973). These three species occasionally manifest a pioneer nature (García et al., 2014; Garcia‐Cervigon et al., 2017).

The taxonomic differences between *J. phoenicea s.s*., *J. turbinata,* and *J. canariensis* may be the result of their divergent evolutionary story from the moment they split from their ancestor in the Oligocene (Mao et al., 2010). This process took place in Europe (Lebreton & Rivera, 1989; Mao et al., 2010). However, the paleodata needed to confirm this hypothesis are scarce (Kvaček, 2002; Stockey et al., 2005). The time of divergence of *J. phoenicea s.s*., *J. turbinata,* and *J. canariensis* has not yet been defined but a relatively early split between *J. phoenicea s.s*. and *J. turbinata* has been suggested (Adams & Schwarzbach, 2013). The further divergence and formation of *J. canariensis* likely coincided with the formation of the Canary Islands. The oldest contemporary existing islands from the archipelago are Fuerteventura and Lanzarote, which started to appear during the Miocene, while the youngest one, El Hierro, did so at the turn of the Pliocene/Pleistocene (Fernández‐Palacios et al., 2011). A dozen or so other islands existed, the oldest dating from the Paleogene, but they subsequently disappeared in accordance with the oceanic island cycle (Whittaker et al., 2007). However, some of the contemporary underwater islets were above sea level during past cold cycles of the Pliocene and the Pleistocene (Fernández‐Palacios et al., 2008, 2011). In view of the above, the *J. canariensis* split from the ancestor probably took place no earlier than in the Miocene.

The current geographic ranges of *Juniperus phoenicea s.s*., *J. turbinata,* and *J. canariensis* evolved under varied climatic conditions (Fernández‐Palacios et al., 2008; Rivas‐Martínez et al., 2004). The adaptation to local conditions in different parts of the ancestral geographic distribution, the subsequent spatial isolation between areas of the current species, and their different pollination phenology (Arista et al., 1997; Romo et al., 2019) are important for taxa differentiation. Thus, we hypothesize that the current ecological niches of *J. phoenicea s.s*., *J. canariensis,* and *J. turbinata* are determined by different climatic conditions, which implies a diversified and species‐specific reaction to global climate change. Our aim was to verify this hypothesis by modeling the current distribution data. Additionally, *J. turbinata* appears to be genetically (Sánchez‐Gómez et al., 2018) and morphologically (Mazur et al., 2018) differentiated into four population groups (see below).

The aims of the present study were 1) to describe the current ecological niches of *J. phoenicea s.s*., *J. turbinata,* and *J. canariensis*, and 2) determine the climatic conditions in their current range. On the basis of these data, additional aims were to define the 3) retrospective and 4) prospective potential niches of each species. The latter included an evaluation of the potential impact of global change on the future geographic ranges of each species according to two scenarios of climate warming. The same procedures were used for the analyses of the four geographic groups of *J. turbinata*.

## 2 MATERIALS AND METHODS

### 2.1 Data

Data on the occurrence of the *J. phoenicea* complex were obtained from the Global Biodiversity Information Facility (GBIF.org), the literature, herbaria, and the authors’ field notes. The data originally did not distinguish *J. phoenicea s.s* from *J. turbinata,* and thus, taxa were segregated using published results of biochemical (Lebreton & Pérez de Paz, 2001; Lebreton & Rivera, 1989), genetic (Adams et al., 2002, 2009, 2010, 2013, 2014; Boratyński et al., 2009; Dzialuk et al., 2011; Jiménez et al., 2017; Sánchez‐Gómez et al., 2018), and biometric (Mazur et al., 2010, 2016, 2018) research. Additionally, their taxonomic status was reviewed according to geographic and ecological criteria. All data were carefully verified to eliminate possible outliers. For Andalusia, the data regarding *J. phoenicea s.s*. and *J. turbinata* distribution were included after reviewing herbaria and the literature, in which these two species were clearly distinguished (Cabezudo et al., 2003; Díez‐Garretas et al., 1996; Pérez Latorre & Cabezudo, 2009; Pérez Latorre et al., 2006, 2014).

We examined the realized, retrospective, and predicted niches separately for *J. phoenicea s.s*., *J. turbinata,* and *J. canariensis* and for the entire *J. phoenicea* complex. Additionally, we analyzed separately the data from the groups of localities of *J. turbinata* detected in the genetic study (Sánchez‐Gómez et al., 2018:7, Figure 2), namely (I) from the Atlantic, African coast and from Europe, eastward to Almeria in Spain (TURAT), (II) central Mediterranean (TURCM), and (III) eastern Mediterranean (TUREM). To these three groups we added a fourth one (IV) from the Arabian Peninsula, the southeastern most group (TURAR).

**FIGURE 1:**
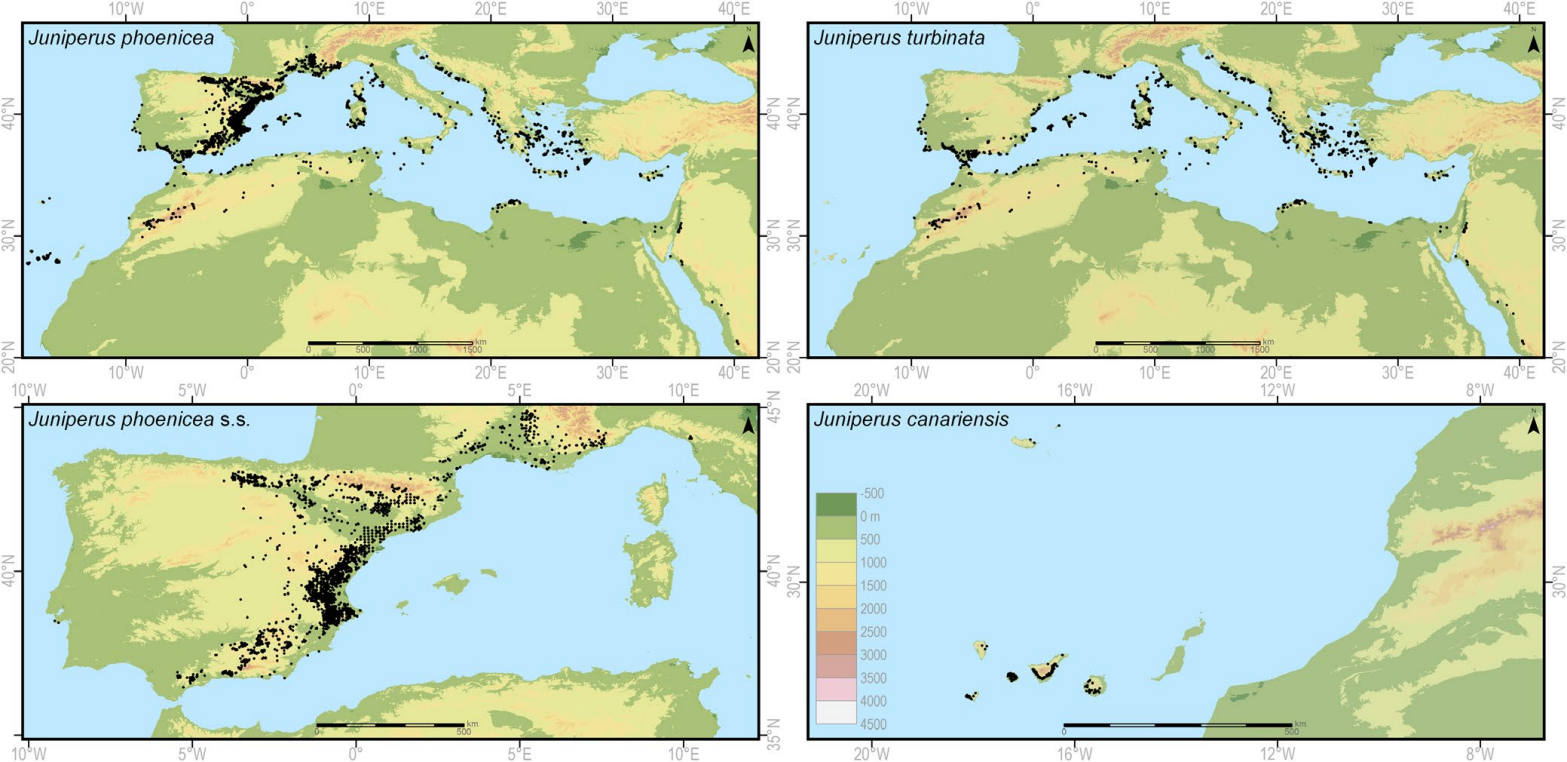
Geographic distribution of *Juniperus phoenicea* complex, *J. phoenicea* s.s., *J. turbinata,* and *J. canariensis* on the background of the mountain systems

Latitude, longitude, and elevation for each locality were obtained from the source data; when this was not possible, they were retrieved from Google Earth. Localities with insufficiently precise descriptions were excluded from the analyses. In total, we gathered more than 10,000 location data, although the majority of them replicated the same information. From this set of data, we selected an exact and precise description for 4,852 localities: 3,254 for *J. phoenicea*, 1,303 for *J. turbinata*, and 295 for *J. canariensis*, respectively (Figure 1). For *J. turbinata*, the numbers of localities were 529, 38, 404, and 32 for TURAT, TURCM, TUREM, and TURAR, respectively (Table S1).

### 2.2 Environmental variables

Temperature, precipitation, and altitude data have been identified as the most influential elements for current ecological niches (Bradie & Leung, 2017). In this study, however, altitude was not used in the models as we did not have these data for all projections. We built ENMs with 19 bioclimatic variables (Table 1; O’Donnell & Ignizio, 2012) and occurrences described by latitude and longitude. We decided to analyze the entire set of bioclimatic data with the aim of detecting differences between the factors determining potential niches of *J. phoenicea*, *J. turbinata,* and *J. canariensis*. We expected that the use of a broad set of climate data would help to respond to the question of adaptation of each species to different bioclimatic conditions, despite their characteristic occurrence in the Mediterranean‐type climate (Rivas‐Martínez et al., 2004).

**TABLE 1:** Contribution [%] of average bioclimatic variables for years 1970–2000 and altitude for realized habitat suitable for *Juniperus phoenicea* complex (ENT), *J. phoenicea s.s*. (PHO), *J. canariensis* (CAN), and *J. turbinata* (TUR); four different parts of *J. turbinata's* geographic range: Atlantic (TURAT), central and west Mediterranean (TURCM), east Mediterranean (TUREM), and the Arabian Peninsula (TURAR)

Bioclimatic factor	ENT	PHO	CAN	TUR	TURAT	TURCM	TUREM	TURAR
BIO1 = Annual Mean Temperature	0.2	0	**10.7**	0.1	0	0.1	0.4	0
BIO2 = Mean Diurnal Range [Mean of monthly (maxT‐minT)]	0.3	**9.4**	1.7	5.8	0.2	**13.3**	**46.6**	3.4
BIO3 = Isothermality (BIO2/BIO7) (* 100)	0.2	1.7	5.7	2.4	2.4	0.5	0.7	1.7
BIO4 = Temperature Seasonality (standard deviation *100)	**21.3**	5.7	**10.3**	1.8	0	0.6	1.3	6.9
BIO5 = Max Temperature of Warmest Month	3.2	2	0	3.8	**21**	0	1.1	**22.7**
BIO6 = Min Temperature of Coldest Month	0.3	2	0	0.3	1.1	**11.9**	6.2	1.3
BIO7 = Temperature Annual Range (BIO5‐BIO6)	1.4	1	0.2	**28.7**	0.3	**39.1**	**11.4**	**17.5**
BIO8 = Mean Temperature of Wettest Quarter	0	0.3	0.6	0.5	0	2.4	1.7	1.6
BIO9 = Mean Temperature of Driest Quarter	0	0.9	0	0.9	0	3.7	0.2	0
BIO10 = Mean Temperature of Warmest Quarter	0	1	0.3	0.2	0.1	0	0	0.1
BIO11 = Mean Temperature of Coldest Quarter	5.7	0.1	0.1	0.9	0.1	9.8	0.5	0.4
BIO12 = Annual Precipitation	**52.7**	0.4	**34.4**	**15.7**	0	0.1	0	**20.9**
BIO13 = Precipitation of Wettest Month	0.1	**15.4**	**15.8**	0.2	0	0.8	2.4	0.1
BIO14 = Precipitation of Driest Month	1.3	6.9	0.4	**18.5**	**20.2**	8	0.5	2.2
BIO15 = Precipitation Seasonality (Coefficient of Variation)	1.5	9.5	4.4	1.2	14	8.4	**15.6**	8.5
BIO16 = Precipitation of Wettest Quarter	2.1	0	4.9	11.3	9.8	0.2	1.5	0.1
BIO17 = Precipitation of Driest Quarter	0.6	**9.7**	1.1	1.3	2	0.3	0.4	1.7
BIO18 = Precipitation of Warmest Quarter	4.5	3.3	**9.3**	0.7	**21.8**	0.4	4	5.7
BIO19 = Precipitation of Coldest Quarter	4.6	**30.9**	0	5.7	6.9	0.3	5.5	5.1

The bioclimatic variables were obtained from the WorldClim 2.1 (WC) database (http://worldclim.org/; Fick & Hijmans, 2017) and Paleoclim (PC) (http://www.paleoclim.org/) (Brown et al., 2018) and had a spatial resolution of 30 arc‐seconds (~1 km). To delineate potential niches during the Last Interglacial period (LIG 120–140 ka BP), WC used the climate data from the CAPE project (Otto‐Bliesner et al., 2006) and data from the Community Climate System Model (CCSM, Gent et al., 2011). For the Last Glacial Maximum climate (LGM, 21 ka BP), PC used the Climatologies at high resolution for the Earth's land surface areas (CHELSA) algorithm on Paleoclimate Modelling Intercomparison Project 3 (PMIP3) data and these were upscaled and masked to sea level. For the Mid‐Holocene climatic optimum (MH, 6 ka BP), WC used CCSM4 and the data were downscaled and calibrated using WorldClim 1.4 software. For the current climate (average for the years 1970–2000), we downloaded bioclimatic variables from the WorldClim 2.1 database (Fick & Hijmans, 2017). For future predictions, we used scenarios of climate change with two representative concentration pathways (RCPs), RCP 2.6 and RCP 8.5 (Collins et al., 2013). The first predicts an increase in radiative forcing by 2.6 W/m^2^ and an increase in temperature of 1°C before 2070 (average for 2061–2080), and the second by 8.5 W/m^2^ and 2°C during the same period. Both are climate projections from GCMs that were downscaled and calibrated using WorldClim 1.4 as the baseline climate.

### 2.3 Ecological niche modeling

The climate data related to species localities were used for predictions of niches by implementation of Maxent 3.4.1. (Phillips, 2017; Phillips et al., 2020). Maximum entropy modeling was used to estimate species probability distributions outside their known area of distribution (Raffini et al., 2020; Yan et al., 2020). Firstly, we evaluated the model with ENMeval R software (Ancillotto et al., 2020; Muscarella et al., 2014). We used 10 k‐fold spatial partitions for each species presence record and evaluated models with the following feature classes: linear, quadratic, hinge, product and threshold, and the following values of regularization multipliers: 0.5, 1, 1.5, 2, 2.5, 3, 3.5, and 4 (Table S2). The parameters used in Maxent were as follows: different features and regularization, bias file, maximum number of iterations at 1,000, convergence threshold 10^–5^, with 10 replicates, cloglog output format, number of background points at 10,000, and replicated run type as cross‐validate.

Receiver Operating Characteristic (ROC) curves were used to evaluate the results of models (Mas et al., 2013; Wang et al., 2007). Area Under the Curve (AUC) values below 0.6 indicated that the results of the predictions were close to random, while 1.0 showed excellent predictions (Table S2). These procedures were conducted for (1) *J. phoenicea* s.s. (PHO), *J. turbinata* (TUR), and *J. canariensis* (CAN), (2) the entire dataset (ENT), and (3) the four groups of genetically and morphologically distinct *J. turbinata* with different geographic ranges: Atlantic–western Mediterranean (TURAT), central Mediterranean (TURCM), eastern Mediterranean (TUREM), and Arabian Peninsula (TURAR).

The predictions of potential niches and climatic variables were mapped using ArcGis Desktop 10.7 and ArcGis Pro 2.3 (ESRI, 2018). Species response curves were drawn to explore the relationship between target species’ habitat suitability. The species distribution chart had values ranging from 0 to 1. These values were grouped into four intervals: unsuitable (<0.2), low (0.2–0.4), medium (0.4–0.6), and high potential (0.6–1) (Yang et al., 2013). We calculated the potential distribution area of the *Juniperus phoenicea* complex based on high‐potential niches (0.6–1).

Retrospective modeling of ecological niches is possible using subfossil materials, but pollen grains of junipers have not been determined to species level (Carrión, 2002; Carrión et al., 2001) and paleo macroremnants are exceptionally rare (Kvaček, 2002; Palamarev, 1989; Palamarev et al., 2005; Stockey et al., 2005; Velitzelos et al., 2014). Junipers evolved in arid environments, a circumstance that notably reduced (or may have completely precluded) macrofossil conservation (Willis & McElwain, 2002).

## 3 RESULTS

### 3.1 Present niche range (1970–2020)

**FIGURE 2:**
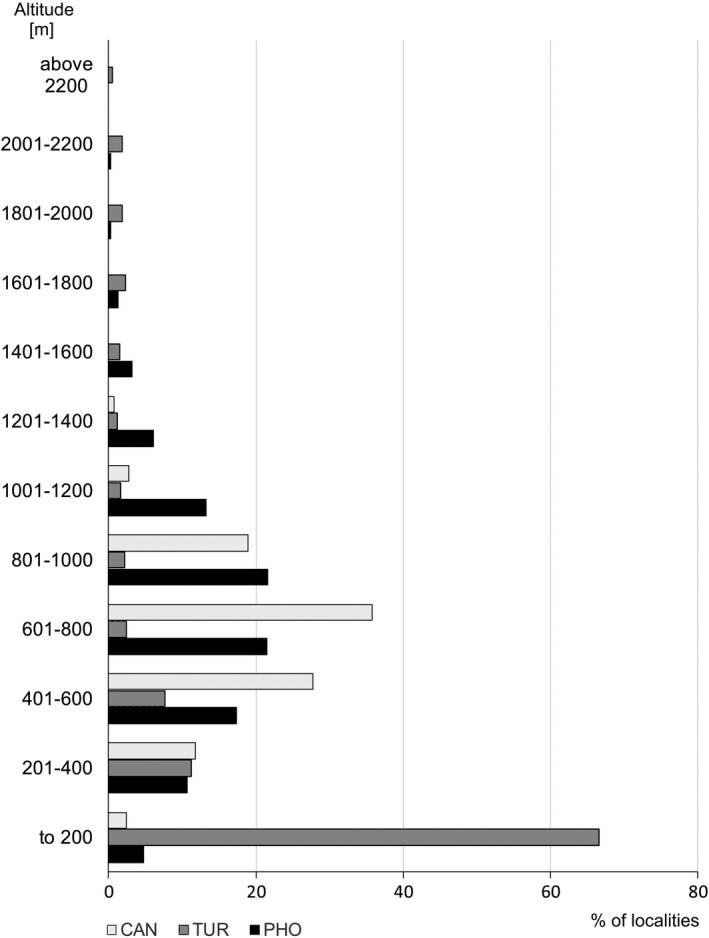
Percentage of georeferenced localities of *Juniperus canariensis* (CAN), *J. turbinata* (TUR), and *J. phoenicea s.s*. (PHO) depending on elevation

The geographic range of *J. phoenicea s.s*. covers the Iberian Peninsula and southeastern France, with several localities in the northwest of Italy (Figure 1). Some existing localities are outside the potential distribution niche, or in areas determined as having low or medium environmental suitability (below 0.6). A case in point is the rocky slopes of the Cabo de Espichel in Portugal and the western Alps. The species occurs at elevations ranging from 50–100 m to about 1600–1800 m, with the highest number of localities between 400 and 1,200 m (Figure 2). Its realized niche is determined mostly by precipitation during the coldest quarter (BIO19) (Table 1), which is low, and by precipitation in the wettest month (BIO 13), which is almost 200 mm (Table S3). Temperature‐defined climatic factors (BIO1–BIO11) explain about 24% of the current geographic range while precipitation factors (BIO12–BIO19) explain over 75%. The jackknife of AUC for *J. phoenicea* confirmed these findings and showed that precipitation in the coldest quarter (BIO18) is the most effective variable for predicting distribution (Fig. S1).

The geographic range of *Juniperus turbinata* extends to the Mediterranean islands, along the shores around the Mediterranean Sea, and along the Atlantic shore of southern Europe and northern Africa. The species enters some inland regions in southern Iberian Peninsula and Sardinia in Europe, the Atlas Mountains in northwest Africa, and mountains of the Arabian Peninsula in southwest Asia, at its southern distribution limit (Figure 1). Several well‐documented localities remain outside areas with suitable environmental conditions for the species in the mountains of northern Africa and in the Arabian Peninsula (Figure 1 and Figure 4). *Juniperus turbinata* has a broader altitudinal range that extends from 0 to 2,410 m; however, in most of the localities it was reported at elevations below 400 m (Figure 2). Its realized niche is determined mostly by the amplitude of annual temperatures (BIO7), by annual precipitation, and by precipitation in the driest month (Table 1). Precipitation‐associated climate factors are responsible for about 55% of the potential niche delimitations, while temperature factors are responsible for over 45%. The jackknife of AUC, however, found that temperature factors are more important in determining the current niche, with annual temperature range and annual mean temperature (BIO7 and BIO1, respectively) having the strongest influence on distribution (Fig. S2).

*Juniperus canariensis* has a narrow geographic range, being found only on the Canary Islands (excluding Lanzarote and Fuerteventura) and Madeira and Porto Santo islands (Figure 1). These localities provide a good reflection of the realized niche, which goes slightly beyond the Canary Islands. The species altitudinal range extends from about 100 to 1,400 m, with about 80% of localities being between 400 and 1,000 m (Figure 2). The potential niche of the species is determined mostly by annual precipitation and precipitation in the wettest month (BIO12 and BIO13, respectively; Table 1). Precipitation‐associated bioclimatic factors determine more than 70% of the potential niche of the species. The jackknife of AUC confirmed this, indicating precipitation as the most important variable, especially during the wettest month and the wettest quarter (BIO13 and BIO16, respectively; Fig. S3).

The realized niche of the *J. phoenicea* complex (ENT) is generally determined by annual precipitation (BIO12) and temperature seasonality (BIO4) (Table 1). The jackknife, however, only confirmed the importance of annual precipitation (BIO12) (Fig. S4).

### 3.2 Past and future geographic range

**FIGURE 3:**
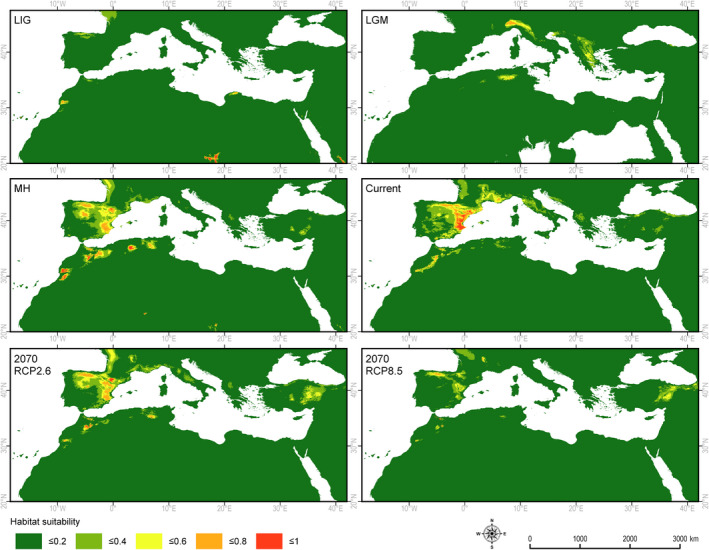
Retrospective, current and prospective climatically determined habitats for *Juniperus phoenicea s.s*.: LIG—Eemian about 125 ka BP, LGM—Last Glacial Maximum about 20 ka BP, MH—Holocene climate optimum about 6,000 BP, Current—current climate conditions, 2070 RCP2.6—optimistic climate warming (2.6 W/m^2^), 2070 RCP8.5—pessimistic climate warming (8.5 W/m^2^)

The retrospective analyses indicate a restricted area not fully suitable for *J. phoenicea s.s*. in Europe during the LIG, based on climate conditions. However, they also identify several places in the mountains of Africa and on Tenerife with suitable climatic conditions. During the LGM, such conditions covered a small area in the western Alps, Apennine Peninsula, and Balkans, while during the MH they covered the whole Iberian Peninsula and the mountains of northern Africa (Figure 3). Those areas with highly suitable climatic conditions were less extensive during the LIG and the LGM than at present. The area of the current potential niche is similar to the one observed during the MH (Table 2).

**TABLE 2:** Estimated areas of potential niches (probability 0.6–1.0) of *Juniperus phoenicea* complex (species codes as in Table 1) during Eemian interstadial (LIG), Late Glacial Maximum (LGM), Holocene climate optimum (MH), and two scenarios of climate change in 2070, compared with the area of the present potential niche

Species	Model	Predicted area [km^2^]	Difference with respect to the present	
			[km^2^]	[%]
ENT	Present	286.163		
	Eemian interstadial about 125 ka BP (LIG)	167.843	−118.320	−41
	Late Glacial Maximum about 22 ka BP (LGM)	286.051	−112	−0
	Holocene climate optimum about 6 ka BP (MH)	252.430	−33.733	−12
	Future‐low climate warming (2.6 W/m^2^)	280.473	−5.690	−2
	Future‐higher climate warming (8.5 W/m^2^)	101.252	−184.911	−65
PHO	Present	76.432		
	Eemian interstadial about 125 ka BP (LIG)	22.539	−53.893	−71
	Late Glacial Maximum about 22 ka BP (LGM)	7.962	−68.470	−90
	Holocene climate optimum about 6 ka BP (MH)	75.746	−686	−1
	Future‐low climate warming (2.6 W/m^2^)	52.275	−24.157	−32
	Future‐higher climate warming (8.5 W/m^2^)	5.949	−70.483	−92
TUR	Present	226.752		
	Eemian interstadial about 125 ka BP (LIG)	95.590	−131.162	−58
	Late Glacial Maximum about 22 ka BP (LGM)	494.383	267.631	+118
	Holocene climate optimum about 6 ka BP (MH)	187.695	−39.057	−17
	Future‐low climate warming (2.6 W/m^2^)	638	−274	−30
	Future‐higher climate warming (8.5 W/m^2^)	105.653	−121.099	−53
CAN	Present	912		
	Eemian interstadial about 125 ka BP (LIG)	0	−912	−100
	Late Glacial Maximum about 22 ka BP (LGM)	0	−912	−100
	Holocene climate optimum about 6 ka BP (MH)	1.197	284	+31
	Future‐low climate warming (2.6 W/m^2^)	197.787	−28.964	−13
	Future‐higher climate warming (8.5 W/m^2^)	370	−543	−59

Bolded values around 10% and more.

**FIGURE 4:**
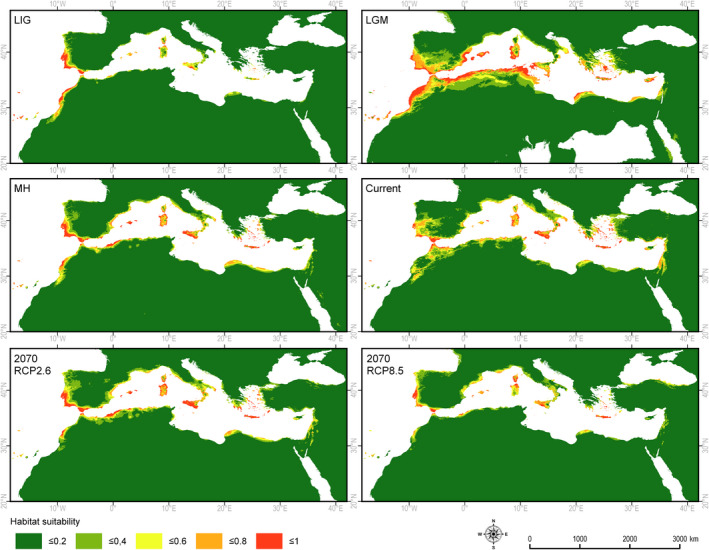
Retrospective, current, and prospective climatically determined habitats for *Juniperus turbinata*: LIG—Eemian about 125 ka BP, LGM—Last Glacial Maximum about 20 ka BP, MH—Holocene climate optimum about 6,000 BP, Current—current climate conditions, 2070 RCP2.6—optimistic climate warming (2.6 W/m^2^), 2070 RCP8.5—deep climate warming (8.5 W/m^2^)

During the LIG, the potential niche of *J. turbinata* covered the Atlantic coast of southern Europe and northern Africa, the Canary Islands, the islands of the Mediterranean Sea (except Cyprus), and some narrow strips along the Mediterranean coast. However, the area with highly suitable climatic conditions (0.6–1.0) was smaller than at present. The LGM period provided the possibility of expansion in the Mediterranean region, the mountains of northern Africa, the Canary Islands and Madeira, and on the Atlantic islands currently present below sea level. The area considered suitable for the species during the LGM was more than twice as large as today, but during the humid period of the Holocene (MH) it was reduced again to a narrower coastal belt and to the islands of the Mediterranean Sea (Figure 4, Table 2).

**FIGURE 5:**
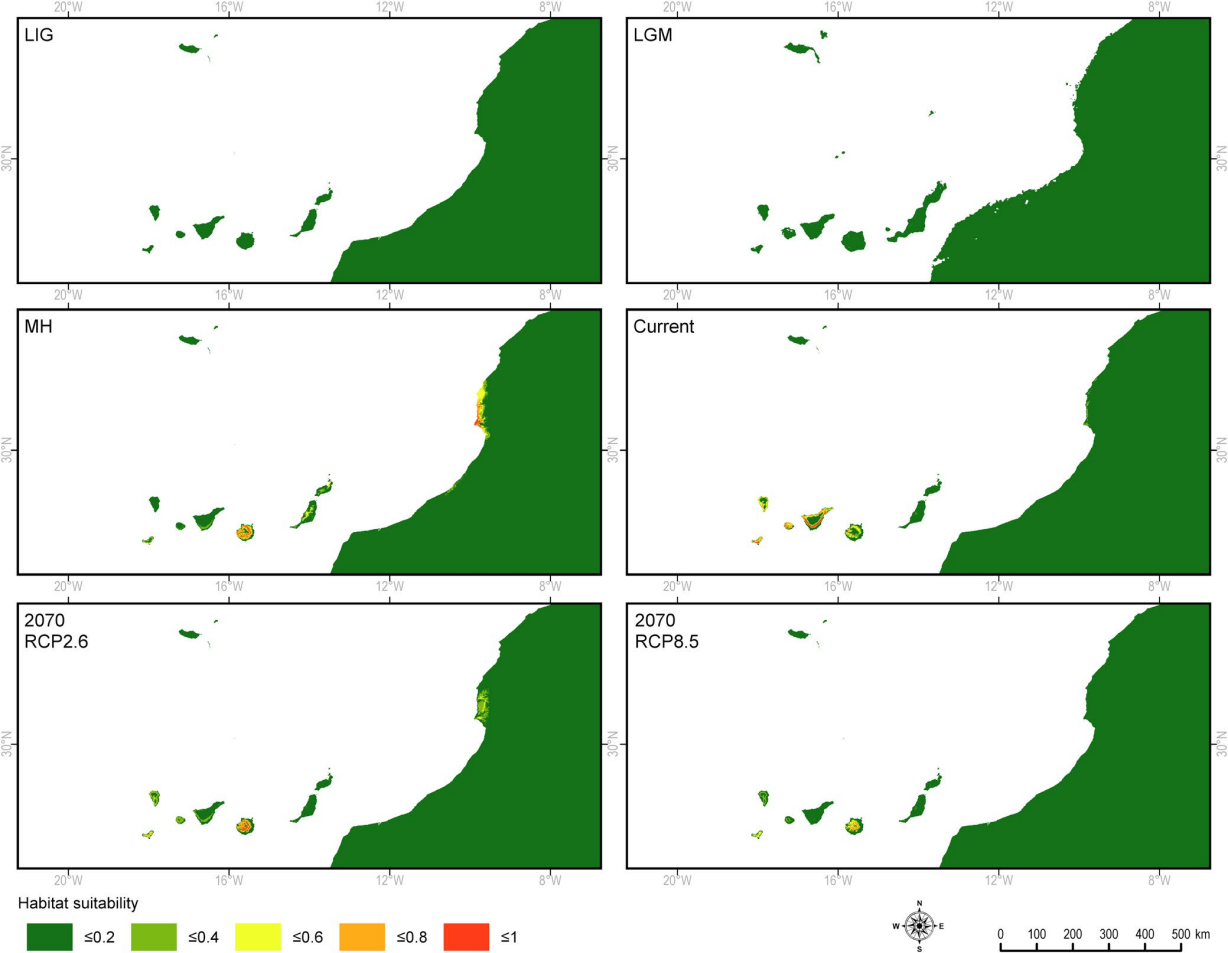
Retrospective, current, and prospective climatically determined habitats for *Juniperus canariensis*: LIG—Eemian about 125 ka BP, LGM—Last Glacial Maximum about 20 ka BP, MH—Holocene climate optimum about 6,000 BP, Current—current climate conditions, 2070 RCP2.6—optimistic climate warming (2.6 W/m^2^), 2070 RCP8.5—pessimistic climate warming (8.5 W/m^2^)

The retrospective analyses did not find suitable habitats for *J. canariensis*, during either the LIG, or the LGM. Climatically suitable habitats appeared during the MH, but only on Gran Canaria Island and on the western shore of North Africa (Tamri region). Interestingly, Lanzarote, Fuerteventura, and the Madeira islands did not have climatic conditions suitable for *J. canariensis* at that time (Figure 5, Table 2).

The complex of *J. phoenicea* (*J. phoenicea s.l*.) seemed to have a broader potential niche during the LIG, LGM, and HM than might be expected from the analyses of niches. The potential niche (0.6–1.0) of the *J. phoenicea* complex during the LIG covered the Atlantic coast of southern Europe and northern Africa, and the Madeira and Canary Islands, with small patches on the Mediterranean islands, but surprisingly, also mountains in the center of the Sahara desert and along the Red Sea. During the LGM, the potential niche covered the Canary Islands, the Atlantic coast of Europe northward to Peniche, the African's coast southward to the region of the Canary Islands, and the Mediterranean region, but with reduced probability in the east. The areas of potential niches during the LGM and the MH were very similar to the current realized niche (Table 2).

Two possible climate change scenarios have been verified, namely RCP2.6 and RCP8.5. In the first, which is more optimistic, the temperature increases by 1°C by 2070. This would reduce the area with a potential climatic niche for each species by varying degrees. In the case of *J. phoenicea s.s*., the potential niche would be reduced to about 70% of the current area and shifted to the mountain regions of the Iberian Peninsula, in northwest Africa, and in less suitable conditions in the west of France, while suitable sites in the southern part of France would disappear. In scenario RCP8.6, only 10% of the current area would retain a climate suitable (0.6–1.0) for the species (Table 2).

The area of the potential niches suitable for *J. turbinata* in the optimistic scenario would be reduced by 30%. This reduction will mostly involve the mountain area in northwest Africa. Compared with the current geographic range of the species, the marginal southern‐most localities in the African and Asian mountain regions would be outside the potential niche. The area of the potential niche in the case of the optimistic scenario will be similar, as detected during the Holocene optimum (Figure 4). The pessimistic climate change scenario would reduce the potential niche area by more than 50% compared with the present (Table 2). The species would suffer a complete loss of suitable climate conditions in the mountain regions. Optimal conditions would be retained in the coastal regions along the Mediterranean Sea and the Atlantic Ocean. Moderately suitable conditions would appear along the Atlantic shore up to France and along the Anatolian shore of the Black Sea (Figure 4).

The potential niche of *J. canariensis* in the optimistic scenario would be only slightly reduced, while in the case of scenario RCP8.5, about 60% of the current area would be lost (Table 2). Suitable climate conditions would only remain on the islands of Gran Canaria and El Hierro, while they would completely disappear from Tenerife, La Palma, Gomera, and the Madeira archipelago (Figure 5).

**FIGURE 6:**
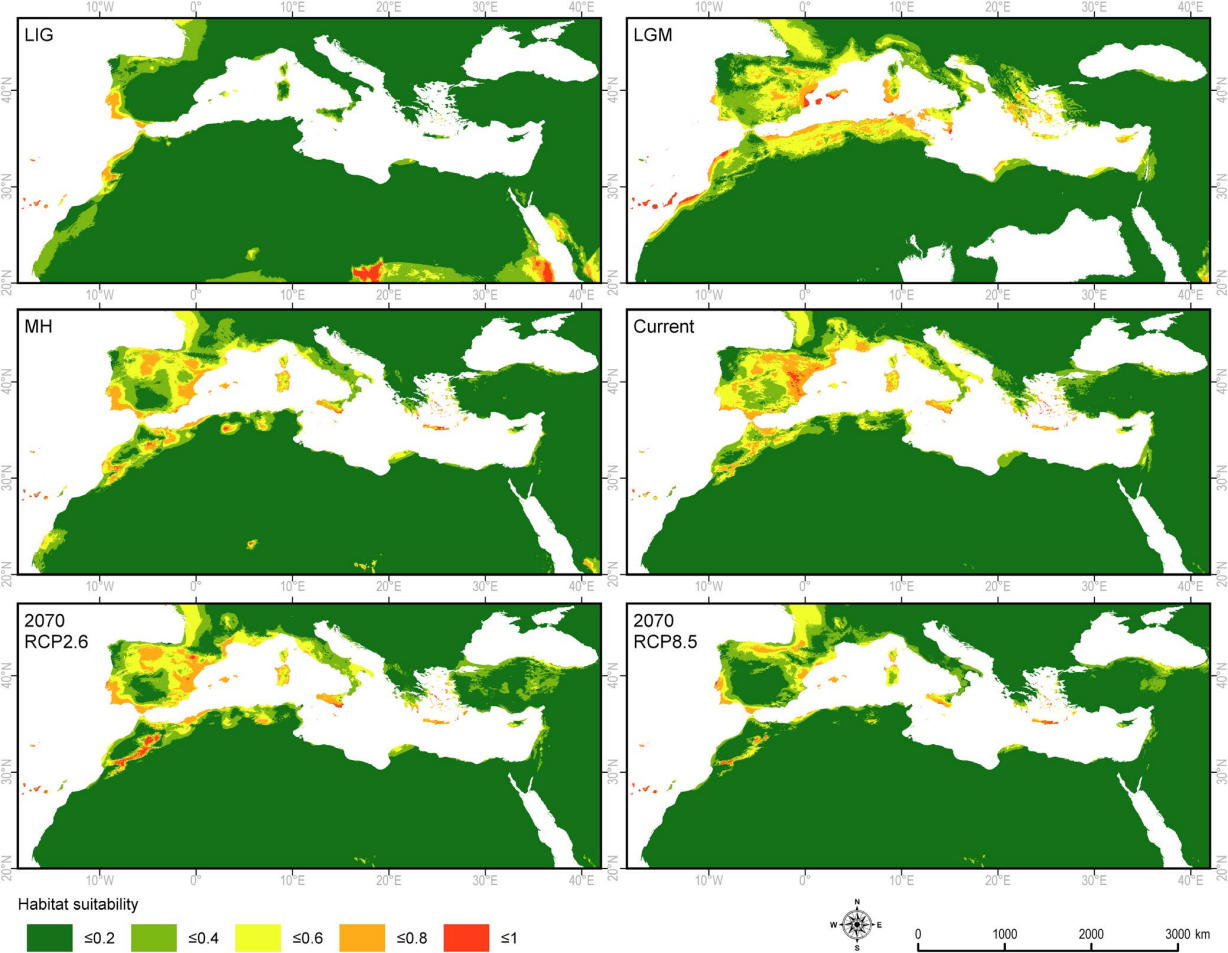
Retrospective, current, and prospective climatically determined habitats for the complex of *Juniperus phoenicea*: LIG—Eemian about 125 ka BP, LGM—Last Glacial Maximum about 20 ka BP, MH—Holocene climate optimum about 6,000 BP, Current—current climate conditions, 2070 RCP2.6—optimistic climate warming (2.6 W/m^2^), 2070 RCP8.5—pessimistic climate warming (8.5 W/m^2^)

The prospective scenario RCP2.6 would reduce the potential niche of the *J. phoenicea* complex mainly in the eastern Mediterranean region, with the main refuge being in the High Atlas in northern Africa and on large Mediterranean islands. The RCP8.5 scenario would drastically reduce its potential niche (Figure 6, Table 2).

The current potential niches of *J. phoenicea*, *J. turbinata,* and *J. canariensis* were determined by a range of variables. The four population groups of *J. turbinata*, the Atlantic‐west Mediterranean (TURAT), central Mediterranean (TURCM), eastern Mediterranean (TUREM), and Arabian (TURAR) also revealed different climatic determinants of their current potential niches (Table 1; Figs. S5‐S8).

## 4 DISCUSSION

Macroremnants of the complex of *J. phoenicea* from past geological periods are scarce or lacking (Kvaček, 2002; Palamarev, 1989; Palamarev et al., 2005; Stockey et al., 2005; Uzquiano & Arnaz, 1997; Velitzelos et al., 2014). Additionally, juniper pollen has not been distinguished to the species level (Carrión et al., 2001). Therefore, we were only able to use the climate conditions of the current realized niche for retrospective and prospective modeling, based on the assumption of a high level of ecological niche conservatism over several geological periods (Rodríguez‐Sánchez & Arroyo, 2008; Svenning et al., 2011; Vessella & Schirone, 2013). Retrospective and prospective modeling of the niches suitable for taxa has been considered a successful tool in various regions of the world (Huntley et al., 1995; Svenning et al., 2011), including the Mediterranean region (Özkan et al., 2015; Rodríguez‐Sánchez & Arroyo, 2008; Romo et al., 2017; Vessella & Schirone, 2013; Walas et al., 2019).

With the aim of verifying the different climatic conditions of potential niches of *J. phoenicea*, *J. turbinata,* and *J. canariensis*, we used an entire set of bioclimatic data, which captures the influence of average and extreme conditions. Extreme conditions may limit physiological processes and restrict species occurrence (Walas et al., 2019). Junipers, which evolved in arid environments (Willis & McElwain, 2002), currently inhabit sites with relatively low levels of precipitation (Adams, 2014; Mao et al., 2010). The taxa of the *J. phoenicea* complex are no exception, occupying areas with a prominent dry period during the warmest months. Thus, the importance of precipitation and bioclimatic factors associated with precipitation for delineation of their current niches is not surprising.

### 4.1 Geographic ranges and their climatic determinants

The three species in the *J. phoenicea* complex have been distinguished from each other during recent decades, and maps of their geographic ranges have been presented either schematically (Adams, 2014; Lebreton & Pérez de Paz, 2001; Lebreton & Rivera, 1989; Mazur et al., 2016) or partially (Otto et al., 2012). The earliest known maps of *J. phoenicea s.l*. were compiled by Jalas & Suominen (1973), Browicz & Zielińki (1982), Boratyński et al. (1992), Charco (2001), and Wazen et al. (2020). The maps showing the distribution of localities (Figure 1) used in the niche modeling constitute the first compilation of the *J. phoenicea* complex distribution.

The main center of occurrence of *Juniperus phoenicea s.s*. covers the eastern part of the Iberian Peninsula, mainly the Ebro Basin, La Mancha, the mountain systems of Andalucía, and southern France. Its populations, even those occurring close to the Mediterranean Sea or the Atlantic coast, grow on limestone mountains and usually at elevations higher than 100–200 m, the maximum elevations being 1,900 m and 1,970 m (Lebreton & Rivera, 1989; Marín Solís, 2019) in the Sierra Nevada and Sierra Mágina, respectively. The distribution map of *J. phoenicea* presented here is comparable with the geographic range proposed by Mazur et al. (2016), but gives details on locality dispersion and altitudinal concentrations. The localities of the species on the Atlantic coast in the Sierra de Arrabida and on Cabo de Espichel are somewhat surprising but are documented in herbarium materials and by morphometric study (Mazur et al., 2018).

The average yearly precipitation in the distribution area of *J. phoenicea s.s*. ranges between 350 and ca 500 mm at lower altitudes but increases to 800–1000 mm in the mountain regions (Lionello et al., 2012; Villar et al., 1997; Walter & Lieth, 1964). The average rainfall in the realized potential niche is 490 mm, with ranges between 300 and 1,100 mm (Table S3). The current distribution of *J. phoenicea* depends mostly on the precipitation falling in the coldest period of the year (December, January, February), which in the center of the species’ distribution on the Iberian Peninsula (Ebro Basin, La Mancha) ranges between 50 and 150 mm, and between 100 and 250 mm in southern France (Lionello et al., 2012).

The species distribution on the Iberian Peninsula is associated with the Mediterranean type of bioclimate, xeric‐oceanic, and pluviseasonal‐oceanic subtype, meso‐, and supra‐Mediterranean thermotype, and semiarid, dry to subhumid ombrotypes (Rivas‐Martínez et al., 2017). The most frequent occurrence of the species is associated with semicontinental to subcontinental bioclimates (Rivas‐Martínez et al., 2017). The typical Phoenician juniper grows in various types of shrubland and light full forest communities, and, being a pioneer tree, colonizes abandoned agricultural lands (García et al., 2014).

In southern France and northern Italy, *J. phoenicea s.s*. occurs in similar climatic conditions to the Mediterranean region, but also grows in temperate regions at specific sites, such as steep, rocky south‐facing slopes in the mountains, or rocky slopes in river ravines (Mandin, 2005). The species is adapted to the Mediterranean climate and to a wide range of bioclimates, from subarid to subhumid or even humid, within the meso‐Mediterranean, supra‐sub‐Mediterranean, and oro‐sub‐Mediterranean zones (Mazur et al., 2016; Rivas‐Martínez et al., 2004).

*Juniperus turbinata* occurs in the Mediterranean region, mainly on the coast and at low elevations up to about 400 m; in the southernmost localities it can be found in the mountains, up to 2,400 m in the High Atlas, and 1,800–2,000 m in southwest Asia (Boratyński et al., 1992; Browicz & Zieliński, 1982; Charco, 2001; El Bana et al., 2010; Kerfoot & Lavranos, 1984; Lebreton & Rivera, 1989; Quézel & Barbero, 1981; Quézel et al., 1994). At coastal sites, *J. turbinata* colonizes maritime dunes and/or rocks, growing on either siliceous or calcium substrata (Ayache et al., 2020; Eliçin 1977; Loidi, 2017; Martinis et al., 2018). It forms populations that can be locally extensive. Inland penetration occurs mostly in maquis and pine or oak forests, and also on the calcareous rocks (Albarreal Núñez & Romero Zarco, 2004; Asensi et al., 2007; Bacchetta, 2006; Capelo et al., 1994; Elmahdy & Mohamed, 2016; Gianguzzi et al., 2012; Martinis et al., 2018; Minissale & Sciandrello, 2013; Quézel & Médail, 2003; Tsiourlis et al., 2016; Vicente Orellana & Galán de Mera, 2019; Zohary, 1973). Some of the southern‐European inland localities are considered as occupying tertiary dunes, uplifted during orogeneses (Hidalgo & Pérez Latorre, 2013; Pérez Latorre et al., 2006).

*Juniperus turbinata* most frequently occurs between sea level and about 400 m, indicating its association with thermo‐Mediterranean climatic conditions and subarid to humid climate ombrotypes (Mazur et al., 2016; Rivas‐Martínez et al., 2004, 2017). The Mediterranean zone where *J. turbinata* grows has average temperatures ranging from 5 to 15°C during winter and 25 to 30°C during summer (Ayache et al., 2020; Calò et al., 2012; Lionello, 2012; Sánchez‐Salguero & Camarero, 2020; Ünal et al., 2003). The absolute minimum winter temperatures rarely fall below 0°C, and the maximum summer temperatures frequently reach 38–40°C (Walter & Lieth, 1964). The annual average precipitation in this belt varies between 400 and 800 mm (Ayache et al., 2020; Elmahdy & Mohamed, 2016; Lionello et al., 2012; Walter & Lieth, 1964), the latter values being recorded in the Mediterranean and Atlantic coastal regions exposed to the west or north, with the direct influence of winds carrying humidity (Lionello et al., 2012). The current potential niche of the species receives an annual average precipitation of more than 600 mm, ranging from 100 to more than 1,250 mm (Table S3).

In the mountains of the Arabian Peninsula and in northwest Africa, *J. turbinata* forms an open woodland/shrubland (Arar et al., 2020; Danin, 1983; El Bana et al., 2010; Kerfoot & Lavranos, 1984; Quézel & Barbero, 1981; Quézel et al., 1994; Quézel & Médail 2003; Zohary, 1973). In the Atlas Mountains, this type of plant community is recognized as transitory between dry thorny oro‐Mediterranean bushlands reminiscent of a pseudosteppe and forest. Here *Juniperus turbinata* grows together with *J. thurifera* L. subsp. *africana* (Maire) Romo & Boratyński and *J. oxycedrus* L., sometimes with *Cupressus atlantica* Gaussen (Quézel & Barbero, 1981; Quézel et al., 1994; Sękiewicz et al., 2014).

The climate conditions of the Atlas Mountains and the mountains of southwest Asia are oro‐Mediterranean in character, with temperatures close to 0°C during winter (Walter & Lieth, 1964; Zohary 1973). The High and Middle Atlas in Morocco receives relatively high precipitation during winter but suffers prolonged drought during late spring and summer (Born et al., 2008; Emberger, 1955). Interestingly, several localities of *J. turbinata* at the top of mountain ridges in northwest Africa remain outside the current potential niche of the species (compare Figures 1 and 4).

The relictual populations of *J. turbinata* on the Sinai Peninsula have survived in an arid region with annual rainfall ca 100 mm and annual mean temperature above 26°C. The species grows here in the so‐called “wetter places,” on rocks on mountain tops, in wadis or at the base of rocks (Danin, 1983; El‐Bana et al., 2010; Moustafa et al., 2016). Currently, however, there is no regeneration of *J. turbinata* (Danin, 1983; Moustafa et al., 2016).

The climatic conditions of most localities of the species in the mountains along the Red Sea have a transitory character between the inland continental desert and the coast. In the mountains, at altitudes between 1,000 and 1,600 m, there is a belt with characteristics resembling the Mediterranean climate, with high temperatures during spring and summer, but wet and cold conditions in winter (Kerfoot & Lavranos, 1984; Palmer, 2013; Schyfsma, 1978; Zohary, 1973).

The potential niches for the four groups of *J. turbinata* (TURAT, TURCM, TUREM, and TURAR) detected during genetic (Sánchez‐Gómez et al., 2018) and morphometric studies (Mazur et al., 2018) appeared to be associated with varying climatic conditions. The differences and the specific response to climate change may reflect their spatial isolation from one another, as is the case with a number of populations of *J. drupacea* (Walas et al., 2019), or even some taxonomic differentiation, as in the case of *Quercus ilex* L. subsp. *ilex* and *Q. ilex* subsp. *ballota* (Desf.) Samp. (López‐Tirado et al., 2018); this hypothesis, however, needs to be tested in further studies.

*Juniperus canariensis* is native to the Canary Islands and the Madeira archipelago, although only isolated specimens are found in the latter (Romo et al., 2019). It does not grow on the driest Canary Islands, Lanzarote, and Fuerteventura, which are exposed to dry and warm winds (Bechtel, 2016; Cropper, 2013). The climate of the Canary Islands is oceanic, with low temperature amplitudes and high humidity, but *J. canariensis* forms homogeneous patches and enters shrub communities in places with relatively low rainfall (Fernández‐Palacios et al., 2008, 2011; Luis González et al., 2017; Otto et al., 2010, 2012; Romo 2018; Romo et al., 2014; Romo & Salvà‐Catarineu, 2013). It grows at elevations mostly between 400 and 1,000 m, higher on the leeward than the windward sides of the islands (Fernández‐Palacios et al., 2008; Otto et al., 2012).

The species distribution in the Macaronesian province is associated with a thermo‐Mediterranean type of bioclimate (Fernández‐Palacios et al., 2008; Rivas‐Martínez et al., 2004) with BIO12, BIO13, and BIO18 being the most influential climate factors. The climate conditions of the current potential niche are characterized by low annual precipitation, which reaches about 340 mm on average and does not go above 420 mm. The lack of rain may be compensated by high air humidity (Fernández‐Palacios et al., 2008; Otto et al., 2010, 2012).

### 4.2 Past and future geographic range

Retrospective analyses indicated the differences between potential climatic niches of *J. phoenicea s.s*. and *J. turbinata* during the LIG and the LGM. Both species had potential niches in northern Africa, but there were potential niches for *J. phoenicea* in the mountains, and for *J. turbinata* mostly along the Atlantic and Mediterranean shores and the Canary Islands. The current spatial isolation of the geographic ranges of *J. phoenicea* and *J. turbinata* supports an early divergence between these two species and their adaptation to different climatic conditions.

High temperatures and low levels of precipitation during May, June, July, and August, and a high diurnal amplitude of temperatures and precipitation seasonality, were identified as important limitations to the current occurrence of *J. phoenicea s.s*. The same limitations would have restricted potential niches during the LIG and the LGM (Jalut et al., 2009; Zucca et al., 2014). The restricted area of the potential niche of *J. phoenicea* during the LIG could also be attributed to annual temperatures that were lower than those currently observed (Allen & Huntley, 2009; Zachos et al., 2001). Taking into account the species’ humidity requirements, their potential LIG niche on Tenerife and close to the African Atlantic shore may be associated with the more humid at that time (Abrantes et al., 2012).

The potential niche distribution of the species during the LGM overlaps with only one refugial area in the Maritime Alps (Médail & Diadema, 2009:1336, Figure 1). Considering the current climate limitations, the enlargement of the potential niche suitable for *J. phoenicea* during the MH could have been a response to the increased precipitation and, to some degree, to the higher temperatures at that time (Jalut et al., 2009; Lionello, 2012; Pérez‐Obiol et al., 2011; Rensen et al., 2012). The higher precipitation created suitable conditions of the species in the Iberian Peninsula at altitudes higher than the ones it currently occupies. The aridification of the Mediterranean climate, which started after the MH (Jalut et al., 1997), has been a reason for the ongoing restriction of *J. phoenicea s.s*. and movement of its potential niche to the more moderate climate zone, where it currently grows.

The overlap between the current potential and realized niches of *J. phoenicea* (Figures 1 and 3) could explain the relatively rapid reaction of the species to past changes in the climate. This can be illustrated by comparing the MH and current potential niches of the species. The reduction in the temperature by approximately 2°C and the fall in precipitation from the MH to the present (Lionello 2012) caused the movement of the species’ potential and realized niches. The expansion of broadleaved trees during the MH could also have contributed to the persistence of *J. phoenicea* at specific rocky sites where there was less competition from broadleaved trees.

The high demands of *J. phoenicea* for access to light, its pioneering character (Asensi et al., 2007; Díez‐Garretas et al., 1996; Franco, 1986; García et al., 2014; Garcia‐Cervigon et al., 2017; Lloret & García, 2016; Lloret & Granzow‐de la Cerda, 2013; Minissale & Sciandrello, 2013), and ornitochorology (Arista et al., 1997; Garcia‐Cervigon et al., 2017) facilitate the rapid colonization of new terrains (García et al., 2014). On the other hand, the relatively long‐life span of the species, especially specimens growing in harsh conditions (Camarero & Ortega‐Martínez, 2019; Mandin, 2005; Mathaux et al., 2016), may have allowed it to persist in some localities even without optimal conditions. Additionally, arbuscular mycorrhizal fungi that are typical of arid and semiarid habitats (Sanguin et al., 2016) may facilitate its persistence. However, the currently existing equilibrium between climate conditions and the occurrence of *J. phoenicea* could be easily disrupted by climate change, mainly by changes in BIO19 and other factors associated with precipitation. The predicted changes in the optimistic scenario (RCP2.6) theoretically diminish the potential niche of the species by more than 30%. The RCP8.5 scenario, in which temperatures would increase by 2°C and precipitation would decrease by 10%–20% in winter and by 30%–40% in summer (Collins et al., 2013), would restrict the potential niche by more than 90%. The reduction in winter precipitation would have a particularly strong influence on the decline in its geographic range (Table 1). Despite this decline, *J. phoenicea* is relatively tolerant to high temperatures and aridity and would survive in specific microsites inaccessible to broadleaved tree formations. Nevertheless, such a drastic reduction in potential niche area would make this species severely endangered.

To a large extent, the current potential and realized niches of *J. turbinata* overlap. The concentration of the potential niche of *J. turbinata* along the coast of Atlantic and on the Mediterranean islands in the LIG resulted from quite high demands for humidity, which was higher there than in the eastern Mediterranean region (Abrantes et al., 2012). The lack of niches suitable for the species in the eastern Mediterranean may also have been the result of lower temperatures than in the western Mediterranean (Abrantes et al., 2012; Cheddadi & Khater, 2016).

The potential niche of the species during the LGM covered most of the glacial refugial areas (Médail & Diadema, 2009). Several localities of *J. turbinata* outside the potential niche at the southern limits of the species’ geographic range may be a remnant of their broader distribution during the LGM (Pulliam 2000). Surprisingly, the potential niches in the LGM did not cover the mountains in the southern part of the Arabian Peninsula and Sinai. This may indicate that populations in these localities are relicts from the LIG or earlier interglacial periods. If so, the species should be considered as having features that allowed millennial persistence in areas outside its optimal conditions. It is also possible that the climate oscillations during the last glacial period (Van Andel 2002) positively influenced the persistence of the species at specific microsites. Wadis, tops of rocky ridges, or bases of steep slopes were considered to be microsites that would allow *J. turbinata* to persist in the Sinai Peninsula (Danin 1983; El Bana et al., 2010; Moustafa et al., 2016) and the mountain system of the west Arabian Peninsula (Kerfoot & Lavranos, 1984; Zohary, 1973).

During the MH, the potential niche of *J. turbinata* in the Mediterranean region was especially strong along the Atlantic coast but was somewhat restricted compared with the LGM niches. Its realized niche during that time may have been further reduced due to competition with broadleaved trees, the distribution of which expanded intensively during the Holocene up to the period of the MH (Calò et al., 2012; Pérez‐Obiol et al., 2011). The aridification of the Holocene climate, which started from the MH, in the eastern Mediterranean region (Finné et al., 2011) seems to have stimulated the expansion of the potential niche. This reconstruction is well supported by the pollen diagrams found in southern Sicily (Noti et al., 2009) where only *J. turbinata* and *J. macrocarpa* Sm. grow, the former on the inland paleo‐dunes and the latter strictly on coastal dunes. These pollen diagrams show the abundant presence of juniper about 6,900 years BP, a progressive decline up to the MH, and then a recovery after the MH but to lower levels, coinciding in time with the abundant presence of *Quercus ilex*. However, the same aridification due to human influence in the African part of the potential niche was a reason for the strong reduction in the realized niche, starting from the MH (Jaouadi et al., 2016).

The current occurrence of populations of *J. turbinata* in the Anti‐, High and Middle Atlas and the Algerian mountains may also be determined by specific site conditions on the mountain ridges (Arar et al., 2020). There the species grows on slopes exposed to the north and northwest, that is, to the winds carrying humidity from the Atlantic Ocean or the Mediterranean Sea. As a result, rainfall is higher than at other sites, and the deposition of dewdrops during the night may also compensate for the water deficit in the summer (Emberger, 1955).

The reduction in the potential niche of *J. turbinata* is more apparent in the pessimistic scenario RCP8.5, in which the temperature would increase by approximately 2°C, precipitation would decrease and there would be a general increase in climate aridity (Allen et al., 2010; Born et al., 2008; Díez‐Garretas et al., 2019; Giannakopoulos et al., 2009; Giorgi & Lionello, 2008; Panagiotis et al., 2013; Paparrizos et al., 2016; Türkeş 2003).

No potential niche for *J. canariensis* (*p* >.6) was identified during either the LIG or the LGM in the Canary and Madeira archipelagos; however, there was a potential niche for *J. turbinata* during the LIG and LGM and for *J. phoenicea s.s* during the LIG. Niches suitable for *J. canariensis* (probability 0.6–1.0) appeared during the MH on Gran Canaria and on the Atlantic coast of Africa north of Agadir, and also on Fuerteventura. From the start of the Holocene about 10,000 years ago, the pollen of *Juniperus* was reported in La Gomera (Nogué et al., 2013). On the other hand, the lack of potential niches *of J. canariensis* during the LIG and the LGM would not rule out its presence in places with more suitable site conditions, as occurred in glacial microrefugia of European trees (Bhagwat & Willis, 2008; Magri, 2008). *Juniperus canariensis* has a lower level of genetic diversity than other species of the *J. phoenicea* complex (Jiménez et al., 2017; Sánchez‐Gómez et al., 2018), but it is still high and comparable to that of other conifers (Boratyński et al., 2014; Bou Dagher‐Kharrat et al., 2007; Conord et al., 2012; Juan et al., 2012). This level of genetic diversity of the Canarian juniper may have been sufficient to allow it to adapt to changing environmental conditions in the past (Jump & Peñuelas, 2005; Matías & Jump, 2012).

The present potential niche of *J. canariensis* is determined mostly by precipitation (BIO13 and BIO18) and annual mean temperature (BIO1). The species currently grows in areas with annual precipitation between 211 and 415 mm (Table S3), but with relatively high air humidity (higher than the other two species). These climate conditions had a similar effect during the LIG and the LGM.

## 5 THREATS

The predicted increase in temperature and decrease in precipitation (the latter by up to 30%, depending on the region) in the Mediterranean basin (Giorgi & Lionello, 2008) will have a great impact on tree biology. Among the climatic factors, precipitation is considered to be the most important, determining the occurrence of many tree species in the Mediterranean and Macaronesian regions (Allen et al., 2010; Matías & Jump, 2012; Thompson, 2005). In our study, precipitation was found to determine the potential niche ranges of the *J. phoenicea* complex to a very high degree. The predicted reduction in precipitation during spring and summer will increase aridity (Giannakopoulos et al., 2009; Panagiotis et al., 2013; Paparrizos et al., 2016) and will also raise tree mortality (Walas et al., 2019). Drought during the summer period has previously provoked an increase in the rate of direct die‐out of juniper specimens in several regions around the Mediterranean Sea (Berger & Heurteaux, 1985; Elmahdy & Mohamed, 2016; Lloret & Granzow‐de la Cerda, 2013; personal observations).

The lack of rain during spring and summer substantially increases the water deficit and the risk of fire (Fernández‐Palacios et al., 2008; Walas et al., 2019). The risk of fire affects all species in the *J. phoenicea* complex and may also be caused by the accumulation of dry grasses among the forests and woodlands due to the reduction in traditional pasturing (JM Montserrat, personal communication). It thus represents a potential threat (Pausas, 2004). Aridification influences the direct seedling mortality after germination (De Dato et al., 2009) and intensifies inbreeding resulting from a lower level of cross‐pollination (Lloret & García, 2016). Lower seedling recruitment also results from a higher level of seed predation associated with increasing temperatures (Mezquida et al., 2016).

The highest amount of precipitation falls during late autumn and early spring (Walter & Lieth, 1964), and a possible water deficit during winter has been detected as a potential limitation for *J. phoenicea s.s*. physiology (Baquedano & Castillo, 2007). Drought during the cold period and early spring is recognized as a possible cause of *J. phoenicea* mortality (Sánchez‐Salguero & Camarero, 2020). During the summer period, rainfall is low and associated predominantly with storms (Lionello et al., 2012). High temperatures in June, July, and August and high evapotranspiration are responsible for drought during this period, an important limitation on *J. phoenicea* occurrence, as is the high diurnal amplitude of temperatures and the precipitation seasonality (Baquedano & Castillo, 2007; Lloret & García, 2016). In the mountains, the minimum temperature may fall to about −7°C (Table S3); however, this does not influence the potential and realized niches of the species (Table 1).

The potential niche of *J. turbinata* is determined mostly by annual temperature range (BIO7) and precipitation factors (BIO12, 14 and 16). The species’ reaction to the climate warming occurring in the southernmost mountainous part of its geographic range is manifested by a high level of mortality in adult trees and a lack of (or highly reduced) recruitment of new plants (personal observations in the High Atlas). The threat to *J. turbinata* would result from the limited photosynthetic efficiency due to the lack of water during the dry period (summer), the very high temperatures, and possible intensified UV radiation (Álvarez‐Rogel et al., 2007; Rubio‐Casal et al., 2010). In the dunes along seashores, it may also be stressed by saltwater infiltration due to high evapotranspiration and the shortage of rainfall resulting from climate warming (Berger & Heurteaux, 1985). The longevity of this species reported to be up to 130 years or more (Martinis et al., 2018) may extend its persistence, but for a relatively short period, mainly due to the frequent rot of the trunks which increases its’ susceptibility to wind breakage.

The winter and early spring drought that has occurred during recent decades could be an important factor in *J. turbinata* dieback (Arar et al., 2020; Sánchez‐Salguero & Camarero, 2020). The possible limitation of *J. turbinata* occurrence could also be associated with the summer water deficit (Armas et al., 2010).

In this context, the localities of *J. turbinata* on the Sinai Peninsula are the most threatened. Its presence there is conneccted with highly local orographic conditions, with slightly modify the desert climate.

The forecasts of niche reduction and consequently of the distribution range of the *Juniperus phoenicea* complex do not take into account direct anthropic actions such as fires, felling, changes in land use, and forestry with alien species, etc., which in the past have decimated juniper populations, especially *J. turbinata*. In the future, human disturbance could aggravate the effects of the scenarios outlined, especially on populations that grow on sandy soils in coastal environments. Those growing in rocky habitats may be less vulnerable. Only decisive action regarding the active protection and strategies to heighten awareness among local populations on the part of the bodies responsible for environmental and biodiversity will be able to counter the phenomenon of local extinctions caused by anthropic pressure.

## CONFLICT OF INTEREST

The authors declare that they have no conflicts of interest to report.

## AUTHOR CONTRIBUTION

**Montserrat Salvà‐Catarineu:** Conceptualization (equal); Formal analysis (lead); Investigation (equal); Methodology (equal); Project administration (lead); Resources (lead); Software (lead); Validation (equal); Visualization (equal); Writing‐original draft (equal); Writing‐review & editing (equal). **Angel Romo:** Conceptualization (equal); Data curation (equal); Funding acquisition (equal); Writing‐original draft (equal). **Małgorzata Mazur:** Data curation (equal); Formal analysis (equal); Investigation (equal); Supervision (equal); Writing‐original draft (equal). **Monika Zielińska:** Data curation (equal); Investigation (equal); Writing‐original draft (equal). **Pietro Minissale:** Conceptualization (equal); Data curation (equal); Investigation (equal); Writing‐original draft (equal); Writing‐review & editing (equal). **Ali A. Dönmez:** Conceptualization (equal); Data curation (equal); Investigation (equal); Writing‐original draft (equal); Writing‐review & editing (equal). **Krystyna Boratyńska:** Conceptualization (equal); Data curation (equal); Writing‐original draft (equal). **Adam Boratyński:** Conceptualization (lead); Data curation (equal); Investigation (equal); Methodology (equal); Resources (equal); Writing‐original draft (lead); Writing‐review & editing (lead).

## 6 ETHICS STATEMENT

1. This material is the authors' own original work, which has not been previously published elsewhere.
2. The paper is not currently being considered for publication elsewhere.
3. The paper reflects the authors' own research and analysis in a truthful and complete manner.
4. The paper properly credits the meaningful contributions of coauthors and coresearchers.
5. The results are appropriately placed in the context of prior and existing research.
6. All sources used are properly disclosed (correct citation). Literally copying of text must be indicated as such by using quotation marks and giving proper reference.
7. All authors have been personally and actively involved in substantial work leading to the paper and will take public responsibility for its content.

## Supporting information

Supplementary Material

## Data Availability

Data available from the Dataverse Digital Repository: https://doi.org/10.34810/data39(Salvà‐Catarineu, et al, 2021).
