# Supplementary material for: Past, present, and future geographic range of the relict Mediterranean and Macaronesian Juniperus phoenicea complex
Source: Ecol Evol. 2021 Mar 25;11(10):5075–95. doi: 10.1002/ece3.7395 (PMC8131820; doi:10.1002/ece3.7395)
Supplement: Supplementary file 1 — Supplementary Material [file ECE3-11-5075-s001.doc]

Supplementary material

Table S1. Species considered for analysis, with sample size of occurrence before and after review.

| Species | Samples before | Samples model |
| --- | --- | --- |
| *Juniperus phoenicea* *complex* (ENT) | 5061 | 4852 |
| *Juniperus phoenicea* s.s. (PHO) | 3258 | 3254 |
| *Juniperus turbinata* (TUR) | 1506 | 1303 |
| *Juniperus canariensis* (CAN) | 297 | 295 |
| *Juniperus turbinata* (TURAT) Atlantic | 560 | 529 |
| *Juniperus turbinata* (TURCM) central Mediterranean | 458 | 338 |
| *Juniperus turbinata* (TUREM) east Mediterranean | 456 | 404 |
| *Juniperus turbinata* (TURAR) Arab Peninsula | 32 | 32 |

Table S2. Evaluation metrics of Maxent SDM generated by ENMeval for *Juniperus phoenicea complex* in the Mediterranean. The feature classes determine the potential shape of the response curves. Settings show feature class combinations (Features: L = Linear, Q = Quadratic, H = Hinge, P = Product, T = Threshold). Regularization multiplier (rm) determines the penalty for adding parameters to the model. AUC (Area Under the Curve) shown AUC calculated on training data (AUCtrain) and averaged over testing data (AUCtest). AIC (The Akaike Information Criterion) model with the lowest AICc value (i.e. ΔAICc = 0) is considered the best model out of the current suite of models; all models with ΔAICc < 2 are generally considered to have substantial support. Number of non-zero coefficients (nparam).

| Species | Features | RM | AUCtrain | AUCtest | ΔAICc | nparam |
| --- | --- | --- | --- | --- | --- | --- |
| *Juniperus phoenicea* *complex* | L Q H P | 1 | 0.737 | 0.731 | 0.0 | 171 |
| *Juniperus phoenicea* s.s. | L Q H | 1 | 0.724 | 0.717 | 0.0 | 129 |
| *Juniperus turbinata* | L Q H P | 3.5 | 0.876 | 0.873 | 0.0 | 68 |
| *Juniperus canariensis* | H | 2.5 | 0.908 | 0.900 | 0.0 | 46 |
| *Juniperus turbinata* (TURAT) | H | 2 | 0.786 | 0.755 | 0.0 | 42 |
| *Juniperus turbinata* (TURCM) | L Q | 2 | 0.878 | 0.875 | 0.0 | 11 |
| *Juniperus turbinata* (TUREM) | L Q | 1.5 | 0.840 | 0.837 | 0.0 | 11 |
| *Juniperus turbinata* (TURAR) | L Q H P | 0.5 | 0.978 | 0.943 | NA | 46 |

Table S3. Bioclimatic factors for potential niches (suitability 0.6-1.0): modal (M), minimal (MIN) and maximal (MAX) values during Last Interglacial (LIG), Last Glacial Maximum (LGM), Mid Holocene (MH), present (PR), RCP 2.6 and RCP 8.5 scenarios

Juniperus phoenicea

| **Bioclimatic factor** | **LIG** |  |  | **LGM** |  |  | **MH** |  |  |
| --- | --- | --- | --- | --- | --- | --- | --- | --- | --- |
|  | MIN | MAX | M | MIN | MAX | M | MIN | MAX | M |
| BIO1 = Annual Mean Temperature | 3.6 | 19.2 | 13.9 | 4.6 | 10.1 | 7.2 | 7.2 | 17.9 | 12.6 |
| BIO2 = Mean Diurnal Range [Mean of monthly (maxT-minT)] | 7.5 | 14.3 | 11.4 | 8.9 | 11.2 | 9.7 | 8.6 | 15.4 | 11.9 |
| BIO3 = Isothermality (BIO2/BIO7) (*100) | 28.0 | 41.0 | 32.4 | 37.0 | 44.0 | 38.4 | 32.0 | 50.0 | 37.3 |
| BIO4 = Temperature Seasonality (standard deviation *100) | 467.2 | 860.5 | 676.7 | 472.5 | 588.0 | 552.3 | 455.1 | 778.3 | 660.3 |
| BIO5 = Max Temperature of Warmest Month | 18.2 | **40.3** | 33.9 | 17.7 | **24.6** | 20.9 | 23.1 | **35.7** | 30.9 |
| BIO6 = Min Temperature of Coldest Month | **-7.4** | 5.4 | -1.1 | **-6.9** | 0.0 | -4.0 | **-7.8** | 5.5 | -0.6 |
| BIO7 = Temperature Annual Range (BIO5-BIO6) | 20.8 | 39.4 | 34.9 | 23.5 | 26.2 | 25.0 | 24.1 | 37.8 | 31.5 |
| BIO8 = Mean Temperature of Wettest Quarter | -1.5 | 24.6 | 16.5 | 0.6 | 10.2 | 6.9 | 2.1 | 25.0 | 10.9 |
| BIO9 = Mean Temperature of Driest Quarter | 2.4 | 23.4 | 10.5 | -2.5 | 17.5 | 11.2 | 2.5 | 25.8 | 19.7 |
| BIO10 = Mean Temperature of Warmest Quarter | 11.6 | 26.9 | 22.9 | 12.4 | 17.6 | 15.4 | 15.3 | 26.1 | 21.9 |
| BIO11 = Mean Temperature of Coldest Quarter | -2.3 | 11.5 | 5.5 | -2.5 | 3.9 | 0.2 | -0.5 | 11.0 | 5.0 |
| BIO12 = Annual Precipitation | 190.0 | **915.0** | 547.1 | 311.0 | **944.0** | 712.9 | 116.0 | **1057.0** | 486.8 |
| BIO13 = Precipitation of Wettest Month | 57.0 | 271.0 | 191.3 | 44.0 | 108.0 | 83.1 | 42.0 | 201.0 | 68.5 |
| BIO14 = Precipitation of Driest Month | 0.0 | **40.0** | 3.5 | 6.0 | **46.0** | 32.4 | 1.0 | **45.0** | 12.2 |
| BIO15 = Precipitation Seasonality (Coefficient of Variation) | 36.0 | 175.0 | 127.6 | 23.0 | 44.0 | 27.9 | 22.0 | 213.0 | 42.5 |
| BIO16 = Precipitation of Wettest Quarter | 103.0 | 560.0 | 408.7 | 117.0 | 316.0 | 243.1 | 86.0 | 518.0 | 171.1 |
| BIO17 = Precipitation of Driest Quarter | 2.0 | 143.0 | 16.4 | 28.0 | 159.0 | 112.4 | 4.0 | 165.0 | 58.9 |
| BIO18 = Precipitation of Warmest Quarter | 26.0 | 297.0 | 100.8 | 28.0 | 160.0 | 116.1 | 28.0 | 167.0 | 74.8 |
| BIO19 = Precipitation of Coldest Quarter | 3.0 | 309.0 | 51.9 | 92.0 | 243.0 | 150.5 | 7.0 | 411.0 | 122.3 |

| **Bioclimatic factor** | **PR** |  |  | **RCP 2.6** |  |  | **RCP 8.5** |  |  |
| --- | --- | --- | --- | --- | --- | --- | --- | --- | --- |
|  | MIN | MAX | M | MIN | MAX | M | MIN | MAX | M |
| BIO1 = Annual Mean Temperature | 6.3 | 18.2 | 12.7 | 7.8 | 18.5 | 13.1 | 9.5 | 16.6 | 12.4 |
| BIO2 = Mean Diurnal Range [Mean of monthly (maxT-minT)] | 7.8 | 15.1 | 12.1 | 9.1 | 15.2 | 11.4 | 9.3 | 15.2 | 11.5 |
| BIO3 = Isothermality (BIO2/BIO7) (*100) | 34.3 | 44.8 | 40.4 | 34.0 | 46.0 | 38.2 | 33.0 | 47.0 | 38.0 |
| BIO4 = Temperature Seasonality (standard deviation*100) | 509.9 | 743.5 | 634.6 | 486.6 | 780.5 | 623.3 | 530.3 | 852.3 | 614.6 |
| BIO5 = Max Temperature of Warmest Month | 21.4 | **33.7** | 29.7 | 23.1 | **34.7** | 29.6 | 24.8 | **36.2** | 29.5 |
| BIO6 = Min Temperature of Coldest Month | **-7.2** | 5.7 | 0.0 | **-6.4** | 5.8 | 0.2 | **-6.8** | 2.0 | -0.4 |
| BIO7 = Temperature Annual Range (BIO5-BIO6) | 22.3 | 34.8 | 29.8 | 24.1 | 36.7 | 29.4 | 25.1 | 39.0 | 29.9 |
| BIO8 = Mean Temperature of Wettest Quarter | 0.9 | 21.0 | 13.0 | 2.7 | 20.5 | 12.5 | 3.8 | 22.9 | 9.7 |
| BIO9 = Mean Temperature of Driest Quarter | 0.9 | 25.3 | 15.5 | 3.3 | 26.0 | 17.1 | 5.6 | 24.4 | 18.5 |
| BIO10 = Mean Temperature of Warmest Quarter | 13.3 | 25.5 | 21.0 | 15.1 | 26.0 | 21.4 | 17.3 | 24.6 | 20.6 |
| BIO11 = Mean Temperature of Coldest Quarter | -0.6 | 11.7 | 5.5 | -0.7 | 11.7 | 5.6 | 0.1 | 8.4 | 4.9 |
| BIO12 = Annual Precipitation | 299.0 | **1097.0** | 487.5 | 301.0 | **1063.0** | 505.7 | 177.0 | **869.0** | 651.5 |
| BIO13 = Precipitation of Wettest Month | 40.0 | 180.0 | 63.7 | 42.0 | 201.0 | 66.7 | 44.0 | 163.0 | 89.0 |
| BIO14 = Precipitation of Driest Month | 1.0 | **54.0** | 19.4 | 1.0 | **48.0** | 19.6 | 1.0 | **38.0** | 23.8 |
| BIO15 = Precipitation Seasonality (Coefficient of Variation) | 20.2 | 77.8 | 33.9 | 19.0 | 80.0 | 32.8 | 23.0 | 132.0 | 35.7 |
| BIO16 = Precipitation of Wettest Quarter | 106.0 | 528.0 | 165.3 | 106.0 | 531.0 | 172.4 | 109.0 | 420.0 | 233.2 |
| BIO17 = Precipitation of Driest Quarter | 8.0 | 198.0 | 77.7 | 10.0 | 163.0 | 80.4 | 11.0 | 143.0 | 91.1 |
| BIO18 = Precipitation of Warmest Quarter | 24.0 | 203.0 | 90.6 | 24.0 | 187.0 | 89.9 | 28.0 | 171.0 | 103.3 |
| BIO19 = Precipitation of Coldest Quarter | 52.0 | 484.0 | 114.4 | 69.0 | 479.0 | 127.7 | 17.0 | 398.0 | 198.7 |

Table S3, continued

Juniperus turbinata

| **Bioclimatic factor** | **LIG** |  |  | **LGM** |  |  | **MH** |  |  |
| --- | --- | --- | --- | --- | --- | --- | --- | --- | --- |
|  | MIN | MAX | M | MIN | MAX | M | MIN | MAX | M |
| BIO1 = Annual Mean Temperature | 10.6 | 21.0 | 16.5 | 9.0 | 20.9 | 14.5 | 11.0 | 20.4 | 16.2 |
| BIO2 = Mean Diurnal Range [Mean of monthly (maxT-minT)] | 4.5 | 11.7 | 8.5 | 1.2 | 12.1 | 6.5 | 4.5 | 12.7 | 8.2 |
| BIO3 = Isothermality (BIO2/BIO7) (*100) | 20.0 | 45.0 | 35.0 | 19.0 | 45.0 | 35.9 | 22.0 | 46.0 | 33.6 |
| BIO4 = Temperature Seasonality (standard deviation *100) | 295.3 | 750.6 | 559.1 | 128.0 | 693.1 | 391.7 | 249.3 | 708.6 | 553.2 |
| BIO5 = Max Temperature of Warmest Month | 21.3 | **33.4** | 30.4 | 12.1 | **32.7** | 25.0 | 20.6 | **33.0** | 30.0 |
| BIO6 = Min Temperature of Coldest Month | **1.4** | 13.9 | 6.1 | **1.7** | 15.5 | 7.3 | **0.0** | 13.8 | 5.9 |
| BIO7 = Temperature Annual Range (BIO5-BIO6) | 13.7 | 29.5 | 24.3 | 5.1 | 28.3 | 17.7 | 12.1 | 30.8 | 24.2 |
| BIO8 = Mean Temperature of Wettest Quarter | 6.6 | 25.3 | 12.0 | 5.5 | 16.3 | 10.6 | 5.7 | 21.6 | 12.3 |
| BIO9 = Mean Temperature of Driest Quarter | 13.2 | 28.1 | 23.4 | 10.6 | 28.3 | 20.9 | 15.3 | 26.8 | 23.2 |
| BIO10 = Mean Temperature of Warmest Quarter | 16.7 | 28.5 | 24.3 | 11.1 | 28.3 | 21.2 | 16.2 | 27.4 | 23.9 |
| BIO11 = Mean Temperature of Coldest Quarter | 5.8 | 17.2 | 10.1 | 4.6 | 16.0 | 9.9 | 5.5 | 17.1 | 9.9 |
| BIO12 = Annual Precipitation | 156.0 | **1141.0** | 621.3 | 145.0 | **1458.0** | 597.6 | 150.0 | **1175.0** | 633.9 |
| BIO13 = Precipitation of Wettest Month | 30.0 | 206.0 | 110.1 | 26.0 | 315.0 | 117.4 | 31.0 | 239.0 | 104.5 |
| BIO14 = Precipitation of Driest Month | 0.0 | **19.0** | 1.6 | 0.0 | **23.0** | 2.5 | 0.0 | **28.0** | 4.0 |
| BIO15 = Precipitation Seasonality (Coefficient of Variation) | 41.0 | 85.0 | 65.1 | 31.0 | 140.0 | 75.4 | 35.0 | 118.0 | 64.9 |
| BIO16 = Precipitation of Wettest Quarter | 66.0 | 501.0 | 283.6 | 72.0 | 891.0 | 324.3 | 87.0 | 629.0 | 282.5 |
| BIO17 = Precipitation of Driest Quarter | 2.0 | 96.0 | 22.6 | 0.0 | 81.0 | 11.4 | 0.0 | 127.0 | 27.3 |
| BIO18 = Precipitation of Warmest Quarter | 6.0 | 96.0 | 29.4 | 0.0 | 141.0 | 15.4 | 0.0 | 161.0 | 44.9 |
| BIO19 = Precipitation of Coldest Quarter | 51.0 | 501.0 | 257.8 | 56.0 | 891.0 | 299.6 | 69.0 | 595.0 | 246.2 |

| **Bioclimatic factor** | **PR** |  |  | **RCP 2.6** |  |  | **RCP 8.5** |  |  |
| --- | --- | --- | --- | --- | --- | --- | --- | --- | --- |
|  | MIN | MAX | M | MIN | MAX | M | MIN | MAX | M |
| BIO1 = Annual Mean Temperature | 10.9 | 21.1 | 16.5 | 11.3 | 22.0 | 17.7 | 11.3 | 22.4 | 18.4 |
| BIO2 = Mean Diurnal Range [Mean of monthly (maxT-minT)] | 2.7 | 13.0 | 8.9 | 4.5 | 12.6 | 8.2 | 4.5 | 11.7 | 7.6 |
| BIO3 = Isothermality (BIO2/BIO7) (*100) | 18.4 | 46.1 | 37.0 | 23.0 | 47.0 | 35.0 | 22.0 | 46.0 | 33.1 |
| BIO4 = Temperature Seasonality (standard deviation*100) | 195.7 | 723.0 | 555.8 | 224.4 | 681.0 | 529.6 | 227.5 | 692.4 | 543.4 |
| BIO5 = Max Temperature of Warmest Month | 18.0 | **32.9** | 29.7 | 20.6 | **33.4** | 30.3 | 21.9 | **33.7** | 30.9 |
| BIO6 = Min Temperature of Coldest Month | **-0.7** | 17.1 | 5.8 | **0.7** | 14.9 | 7.3 | **2.3** | 15.1 | 8.2 |
| BIO7 = Temperature Annual Range (BIO5-BIO6) | 8.3 | 31.4 | 23.9 | 11.3 | 30.6 | 23.0 | 11.4 | 28.8 | 22.8 |
| BIO8 = Mean Temperature of Wettest Quarter | 5.9 | 21.4 | 12.2 | 6.3 | 22.4 | 13.3 | 6.3 | 21.9 | 14.3 |
| BIO9 = Mean Temperature of Driest Quarter | 14.1 | 27.5 | 23.4 | 15.4 | 28.3 | 24.4 | 16.2 | 29.6 | 25.2 |
| BIO10 = Mean Temperature of Warmest Quarter | 14.8 | 27.8 | 23.6 | 16.2 | 28.4 | 24.7 | 16.7 | 29.6 | 25.6 |
| BIO11 = Mean Temperature of Coldest Quarter | 5.3 | 18.9 | 10.2 | 5.5 | 18.1 | 11.3 | 5.9 | 17.8 | 11.9 |
| BIO12 = Annual Precipitation | 124.0 | **1251.0** | 607.2 | 138.0 | **1219.0** | 571.9 | 183.0 | **1142.0** | 558.9 |
| BIO13 = Precipitation of Wettest Month | 14.0 | 262.0 | 104.1 | 30.0 | 258.0 | 98.0 | 28.0 | 259.0 | 98.3 |
| BIO14 = Precipitation of Driest Month | 0.0 | **35.0** | 4.8 | 0.0 | **28.0** | 4.8 | 0.0 | **30.0** | 5.9 |
| BIO15 = Precipitation Seasonality (Coefficient of Variation) | 18.5 | 120.1 | 67.7 | 28.0 | 115.0 | 66.1 | 28.0 | 113.0 | 66.1 |
| BIO16 = Precipitation of Wettest Quarter | 40.0 | 651.0 | 282.0 | 75.0 | 689.0 | 265.0 | 76.0 | 615.0 | 260.4 |
| BIO17 = Precipitation of Driest Quarter | 0.0 | 178.0 | 27.8 | 0.0 | 113.0 | 27.9 | 0.0 | 147.0 | 32.3 |
| BIO18 = Precipitation of Warmest Quarter | 0.0 | 238.0 | 39.6 | 0.0 | 150.0 | 40.8 | 0.0 | 177.0 | 47.1 |
| BIO19 = Precipitation of Coldest Quarter | 27.0 | 651.0 | 256.2 | 65.0 | 689.0 | 239.6 | 76.0 | 615.0 | 225.4 |

Table S3, continued

Juniperus canariensis

| **Bioclimatic factor** | **LIG** |  |  | **LGM** |  |  | **MH** |  |  |
| --- | --- | --- | --- | --- | --- | --- | --- | --- | --- |
|  | MIN | MAX | M | MIN | MAX | M | MIN | MAX | M |
| BIO1 = Annual Mean Temperature |  |  |  |  |  |  | 14.5 | 18.4 | 16.6 |
| BIO2 = Mean Diurnal Range [Mean of monthly (maxT-minT)] |  |  |  |  |  |  | 6.6 | 9.7 | 7.8 |
| BIO3 = Isothermality (BIO2/BIO7) (*100) |  |  |  |  |  |  | 43.0 | 49.0 | 46.0 |
| BIO4 = Temperature Seasonality (standard deviation *100) |  |  |  |  |  |  | 262.6 | 366.1 | 306.2 |
| BIO5 = Max Temperature of Warmest Month |  |  |  |  |  |  | 23.3 | **27.1** | 25.1 |
| BIO6 = Min Temperature of Coldest Month |  |  |  |  |  |  | **5.5** | 11.7 | 8.3 |
| BIO7 = Temperature Annual Range (BIO5-BIO6) |  |  |  |  |  |  | 14.8 | 20.6 | 16.9 |
| BIO8 = Mean Temperature of Wettest Quarter |  |  |  |  |  |  | 11.7 | 16.2 | 13.9 |
| BIO9 = Mean Temperature of Driest Quarter |  |  |  |  |  |  | 18.2 | 21.7 | 19.9 |
| BIO10 = Mean Temperature of Warmest Quarter |  |  |  |  |  |  | 18.9 | 22.4 | 20.7 |
| BIO11 = Mean Temperature of Coldest Quarter |  |  |  |  |  |  | 10.6 | 15.2 | 12.8 |
| BIO12 = Annual Precipitation |  |  |  |  |  |  | 159.0 | **403.0** | 322.6 |
| BIO13 = Precipitation of Wettest Month |  |  |  |  |  |  | 36.0 | 69.0 | 58.1 |
| BIO14 = Precipitation of Driest Month |  |  |  |  |  |  | 0.0 | **1.0** | 0.5 |
| BIO15 = Precipitation Seasonality (Coefficient of Variation) |  |  |  |  |  |  | 70.0 | 86.0 | 77.7 |
| BIO16 = Precipitation of Wettest Quarter |  |  |  |  |  |  | 89.0 | 188.0 | 161.0 |
| BIO17 = Precipitation of Driest Quarter |  |  |  |  |  |  | 0.0 | 7.0 | 3.7 |
| BIO18 = Precipitation of Warmest Quarter |  |  |  |  |  |  | 10.0 | 21.0 | 14.6 |
| BIO19 = Precipitation of Coldest Quarter |  |  |  |  |  |  | 79.0 | 171.0 | 147.2 |

| **Bioclimatic factor** | **PR** |  |  | **RCP 2.6** |  |  | **RCP 8.5** |  |  |
| --- | --- | --- | --- | --- | --- | --- | --- | --- | --- |
|  | MIN | MAX | M | MIN | MAX | M | MIN | MAX | M |
| BIO1 = Annual Mean Temperature | 15.1 | 19.5 | 17.3 | 14.9 | 18.8 | 17.0 | 15.5 | 18.7 | 17.6 |
| BIO2 = Mean Diurnal Range [Mean of monthly (maxT-minT)] | 5.2 | 9.3 | 7.2 | 6.8 | 7.9 | 7.5 | 6.9 | 8.6 | 7.8 |
| BIO3 = Isothermality (BIO2/BIO7) (*100) | 42.2 | 53.8 | 47.8 | 43.0 | 50.0 | 46.7 | 45.0 | 53.0 | 48.9 |
| BIO4 = Temperature Seasonality (standard deviation*100) | 225.6 | 349.1 | 284.3 | 248.9 | 321.7 | 281.9 | 245.5 | 319.4 | 274.5 |
| BIO5 = Max Temperature of Warmest Month | 23.2 | **28.4** | 25.9 | 24.8 | **27.4** | 26.2 | 25.1 | **27.9** | 26.5 |
| BIO6 = Min Temperature of Coldest Month | **7.6** | 13.6 | 10.8 | **7.4** | 12.3 | 10.2 | **8.3** | 12.2 | 10.6 |
| BIO7 = Temperature Annual Range (BIO5-BIO6) | 11.4 | 18.9 | 15.1 | 14.8 | 17.5 | 16.0 | 14.8 | 17.3 | 15.9 |
| BIO8 = Mean Temperature of Wettest Quarter | 11.5 | 17.7 | 14.8 | 12.0 | 16.7 | 14.6 | 12.8 | 16.5 | 14.9 |
| BIO9 = Mean Temperature of Driest Quarter | 18.0 | 22.4 | 20.3 | 18.8 | 21.7 | 20.3 | 19.2 | 22.3 | 20.7 |
| BIO10 = Mean Temperature of Warmest Quarter | 19.1 | 23.0 | 21.1 | 19.4 | 22.4 | 21.0 | 19.7 | 22.7 | 21.3 |
| BIO11 = Mean Temperature of Coldest Quarter | 11.5 | 16.6 | 14.2 | 11.2 | 15.8 | 13.7 | 11.9 | 15.7 | 14.2 |
| BIO12 = Annual Precipitation | 211.0 | **415.0** | 336.7 | 243.0 | **381.0** | 314.3 | 282.0 | **374.0** | 331.2 |
| BIO13 = Precipitation of Wettest Month | 39.0 | 76.0 | 63.3 | 50.0 | 73.0 | 62.6 | 56.0 | 73.0 | 64.6 |
| BIO14 = Precipitation of Driest Month | 0.0 | **2.0** | 0.6 | 0.0 | **1.0** | 0.9 | 0.0 | **2.0** | 1.5 |
| BIO15 = Precipitation Seasonality (Coefficient of Variation) | 71.2 | 85.0 | 79.2 | 78.0 | 85.0 | 80.9 | 76.0 | 84.0 | 78.4 |
| BIO16 = Precipitation of Wettest Quarter | 107.0 | 208.0 | 169.9 | 135.0 | 206.0 | 171.2 | 152.0 | 199.0 | 177.5 |
| BIO17 = Precipitation of Driest Quarter | 10.0 | 42.0 | 14.3 | 2.0 | 6.0 | 5.0 | 2.0 | 13.0 | 8.9 |
| BIO18 = Precipitation of Warmest Quarter | 10.0 | 42.0 | 14.3 | 12.0 | 17.0 | 14.4 | 13.0 | 22.0 | 18.6 |
| BIO19 = Precipitation of Coldest Quarter | 107.0 | 200.0 | 148.7 | 107.0 | 180.0 | 146.3 | 125.0 | 187.0 | 158.9 |

Table S3, continued

Juniperus phoenicea s.l.

| **Bioclimatic factor** | **LIG** |  |  | **LGM** |  |  | **MH** |  |  |
| --- | --- | --- | --- | --- | --- | --- | --- | --- | --- |
|  | MIN | MAX | M | MIN | MAX | M | MIN | MAX | M |
| BIO1 = Annual Mean Temperature | 7.6 | 24.6 | 17.6 | -0.3 | 20.5 | 13.3 | 6.7 | 20.6 | 14.4 |
| BIO2 = Mean Diurnal Range [Mean of monthly (maxT-minT)] | 4.5 | 14.7 | 9.8 | 1.2 | 16.0 | 6.5 | 4.5 | 16.3 | 9.9 |
| BIO3 = Isothermality (BIO2/BIO7) (*100) | 20.0 | 48.0 | 32.9 | 20.0 | 53.0 | 36.1 | 22.0 | 53.0 | 35.5 |
| BIO4 = Temperature Seasonality (standard deviation *100) | 283.8 | 777.3 | 580.8 | 127.3 | 645.5 | 375.0 | 212.9 | 769.5 | 598.3 |
| BIO5 = Max Temperature of Warmest Month | 21.2 | **42.8** | 35.0 | 2.4 | **32.0** | 23.4 | 19.6 | **36.0** | 30.3 |
| BIO6 = Min Temperature of Coldest Month | -**7.6** | 15.6 | 5.3 | -**6.1** | 15.5 | 6.2 | **-8.9** | 14.4 | 2.8 |
| BIO7 = Temperature Annual Range (BIO5-BIO6) | 13.7 | 39.9 | 29.7 | 5.0 | 32.9 | 17.1 | 12.1 | 39.3 | 27.4 |
| BIO8 = Mean Temperature of Wettest Quarter | 0.9 | 28.5 | 18.6 | -1.7 | 17.2 | 10.1 | 1.1 | 26.8 | 11.9 |
| BIO9 = Mean Temperature of Driest Quarter | 2.1 | 27.7 | 16.0 | 1.5 | 27.1 | 19.1 | 2.0 | 26.7 | 21.1 |
| BIO10 = Mean Temperature of Warmest Quarter | 15.4 | 30.3 | 25.4 | 1.5 | 27.1 | 19.6 | 14.1 | 27.2 | 22.8 |
| BIO11 = Mean Temperature of Coldest Quarter | -1.1 | 19.0 | 10.5 | -2.1 | 16.1 | 8.8 | -1.8 | 17.5 | 7.5 |
| BIO12 = Annual Precipitation | 149.0 | **1002.0** | 581.7 | 74.0 | **1487.0** | 501.6 | 53.0 | **1214.0** | 572.9 |
| BIO13 = Precipitation of Wettest Month | 34.0 | 272.0 | 178.6 | 28.0 | 310.0 | 90.5 | 24.0 | 238.0 | 88.1 |
| BIO14 = Precipitation of Driest Month | 0.0 | **38.0** | 1.1 | 0.0 | **48.0** | 3.5 | 0.0 | **51.0** | 9.2 |
| BIO15 = Precipitation Seasonality (Coefficient of Variation) | 36.0 | 179.0 | 121.8 | 23.0 | 146.0 | 69.3 | 20.0 | 207.0 | 52.9 |
| BIO16 = Precipitation of Wettest Quarter | 77.0 | 563.0 | 406.2 | 67.0 | 854.0 | 251.7 | 33.0 | 627.0 | 229.4 |
| BIO17 = Precipitation of Driest Quarter | 0.0 | 139.0 | 11.2 | 0.0 | 157.0 | 16.5 | 0.0 | 199.0 | 48.1 |
| BIO18 = Precipitation of Warmest Quarter | 6.0 | 328.0 | 79.4 | 0.0 | 160.0 | 20.6 | 4.0 | 244.0 | 65.0 |
| BIO19 = Precipitation of Coldest Quarter | 0.0 | 483.0 | 98.1 | 3.0 | 638.0 | 222.3 | 2.0 | 527.0 | 181.6 |

| **Bioclimatic factor** | **PR** |  |  | **RCP 2.6** |  |  | **RCP 8.5** |  |  |
| --- | --- | --- | --- | --- | --- | --- | --- | --- | --- |
|  | MIN | MAX | M | MIN | MAX | M | MIN | MAX | M |
| BIO1 = Annual Mean Temperature | 5.9 | 21.6 | 14.1 | 4.7 | 21.9 | 15.4 | 4.9 | 22.7 | 17.0 |
| BIO2 = Mean Diurnal Range [Mean of monthly (maxT-minT)] | 2.7 | 15.4 | 10.5 | 4.5 | 17.3 | 10.3 | 4.5 | 17.2 | 9.2 |
| BIO3 = Isothermality (BIO2/BIO7) (*100) | 18.4 | 56.5 | 38.7 | 23.0 | 55.0 | 37.1 | 22.0 | 58.0 | 36.1 |
| BIO4 = Temperature Seasonality (standard deviation*100) | 177.0 | 734.6 | 596.0 | 202.0 | 769.3 | 584.3 | 181.4 | 791.1 | 555.1 |
| BIO5 = Max Temperature of Warmest Month | 19.7 | **34.0** | 29.5 | 20.5 | **36.6** | 30.6 | 20.0 | **37.5** | 31.0 |
| BIO6 = Min Temperature of Coldest Month | **-7.5** | 17.1 | 2.4 | **-11.4** | 16.2 | 3.4 | -**11.6** | 17.1 | 5.9 |
| BIO7 = Temperature Annual Range (BIO5-BIO6) | 8.3 | 34.9 | 27.1 | 11.3 | 40.3 | 27.2 | 11.2 | 41.4 | 25.1 |
| BIO8 = Mean Temperature of Wettest Quarter | 2.4 | 21.2 | 12.0 | -2.4 | 22.9 | 12.4 | 0.5 | 25.1 | 13.1 |
| BIO9 = Mean Temperature of Driest Quarter | 0.1 | 27.0 | 19.8 | 2.9 | 27.8 | 21.9 | 5.8 | 29.6 | 24.0 |
| BIO10 = Mean Temperature of Warmest Quarter | 14.9 | 27.0 | 21.8 | 14.2 | 27.8 | 23.2 | 14.1 | 29.6 | 24.4 |
| BIO11 = Mean Temperature of Coldest Quarter | -1.6 | 19.0 | 7.4 | -3.4 | 19.4 | 8.4 | -3.7 | 20.3 | 10.3 |
| BIO12 = Annual Precipitation | 157.0 | **1085.0** | 533.4 | 104.0 | **1076.0** | 521.2 | 112.0 | **1116.0** | 542.2 |
| BIO13 = Precipitation of Wettest Month | 34.0 | 224.0 | 76.9 | 31.0 | 219.0 | 77.0 | 31.0 | 207.0 | 86.8 |
| BIO14 = Precipitation of Driest Month | 0.0 | **52.0** | 13.3 | 0.0 | **50.0** | 11.3 | 0.0 | **49.0** | 10.2 |
| BIO15 = Precipitation Seasonality (Coefficient of Variation) | 18.8 | 116.9 | 45.9 | 19.0 | 123.0 | 47.6 | 19.0 | 144.0 | 56.9 |
| BIO16 = Precipitation of Wettest Quarter | 90.0 | 588.0 | 205.2 | 63.0 | 564.0 | 205.7 | 67.0 | 511.0 | 231.6 |
| BIO17 = Precipitation of Driest Quarter | 0.0 | 195.0 | 58.8 | 1.0 | 191.0 | 52.5 | 1.0 | 196.0 | 49.1 |
| BIO18 = Precipitation of Warmest Quarter | 3.0 | 215.0 | 68.6 | 2.0 | 191.0 | 62.1 | 2.0 | 196.0 | 59.6 |
| BIO19 = Precipitation of Coldest Quarter | 52.0 | 588.0 | 167.2 | 13.0 | 559.0 | 174.3 | 13.0 | 426.0 | 197.7 |


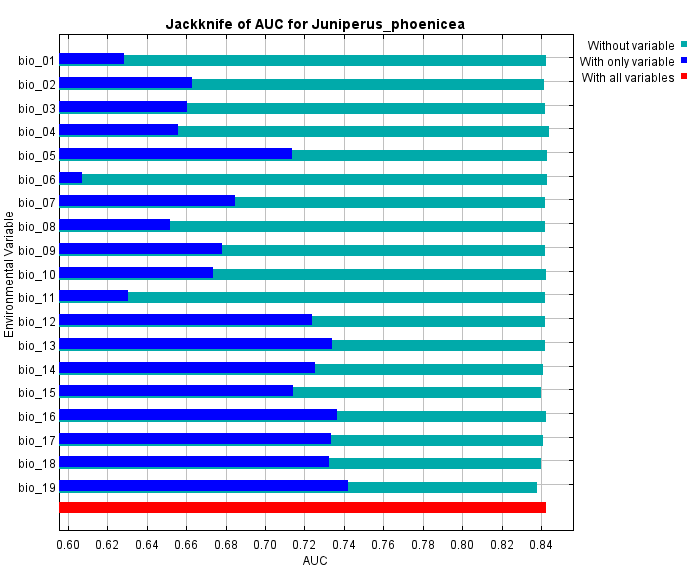


Figure S1. Jackknife test, using AUC (mean AUC=0.842) on test data of *Juniperus phoenicea* s.s. (PHO). The precipitation of coldest quarter variable (bio_19), is the one that generalizes best, is the most important variable to transfer the future distribution under climate change.


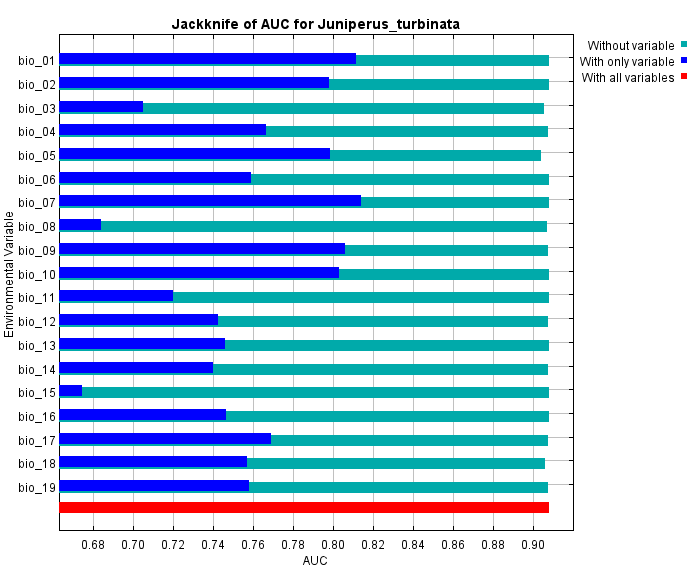


Figure S2. Jackknife test, using AUC (mean AUC=0.908) on test data of *Juniperus turbinata* (TUR). The temperature annual range variable (bio_07), it is the variable that best generalizes, it is the most important variable to transfer past or future distributions under climate change.


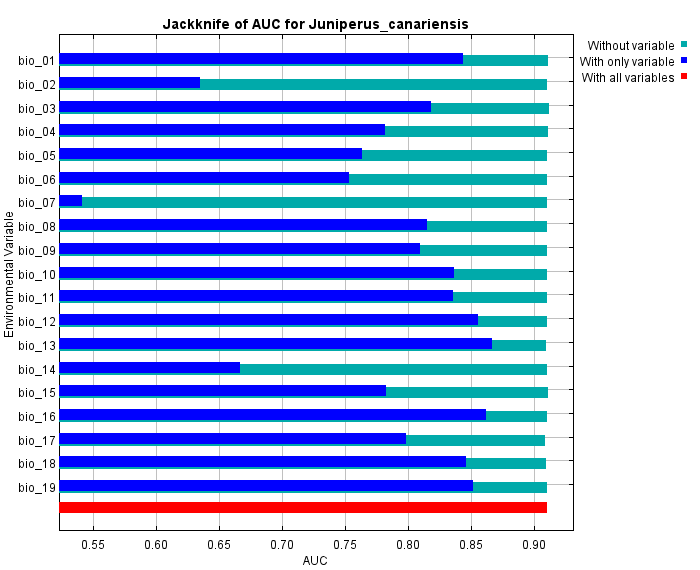


Figure S3. Jackknife test, using AUC (mean AUC=0.911) on test data of *Juniperus canariensis* (CAN). The precipitation of wettest month variable (bio_13), it is the variable that best generalizes, it is the most important variable to transfer past or future distributions under climate change.


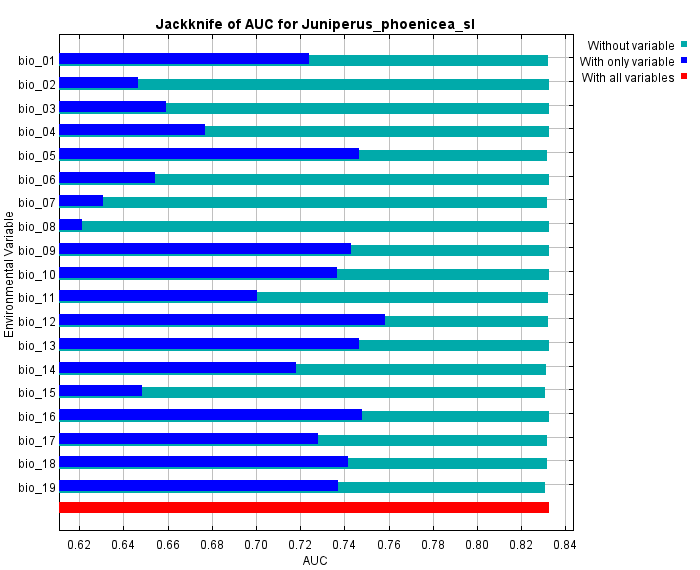


Figure S4. Jackknife test, using AUC (mean AUC=0.883) on test data of *Juniperus phoenicea* *complex* (ENT). The annual precipitation variable (bio_12), is the one that generalizes best, is the most important variable to transfer the future distribution under climate change.


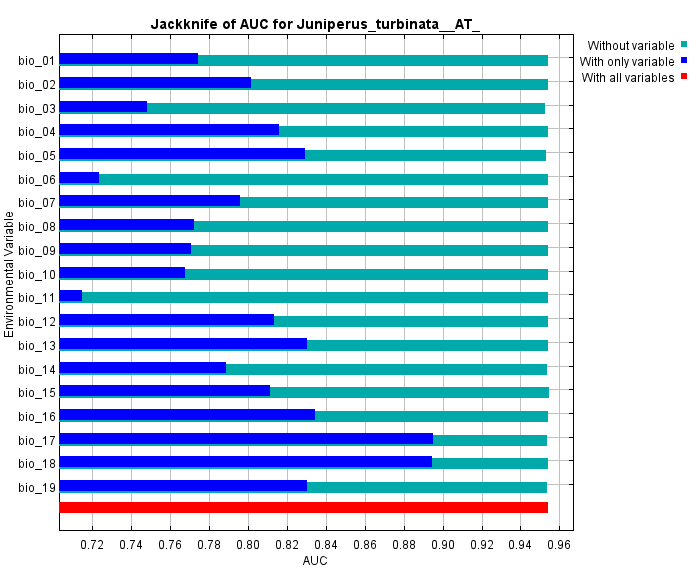


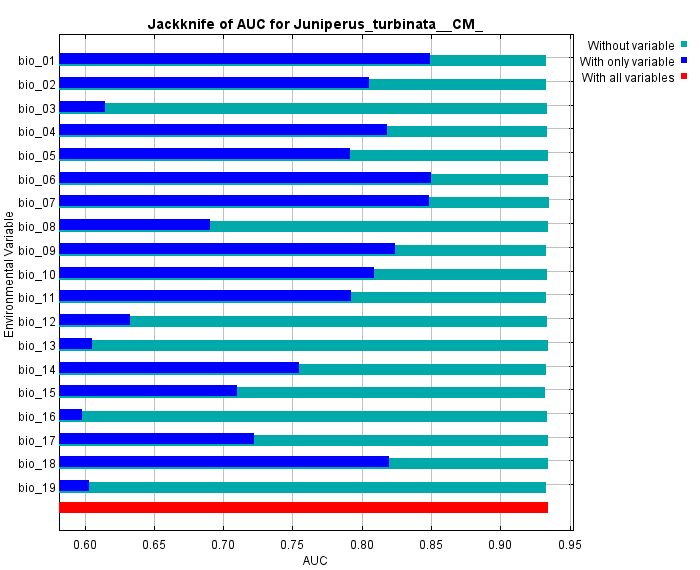
Figure S5. Jackknife test, using AUC (mean AUC=0.954) on test data of Atlantic-west Mediterranean *Juniperus turbinata* (TURAT). The precipitation of driest quarter variable (bio_17) and the precipitation of warmest quarter variable (bio_18), are the variables that generalize best, are the most important variables to transfer past or future distributions under climate change.

Figure S6. Jackknife test, using AUC (mean AUC=0.934) on test data of *Juniperus turbinata* (TURCM). The annual mean temperature variable (bio_01), the mean temperature of coldest month variable (bio_06) and the temperature annual range variable (bio_07), are the variables that generalize best, are the most important variables to transfer past or future distributions under climate change.


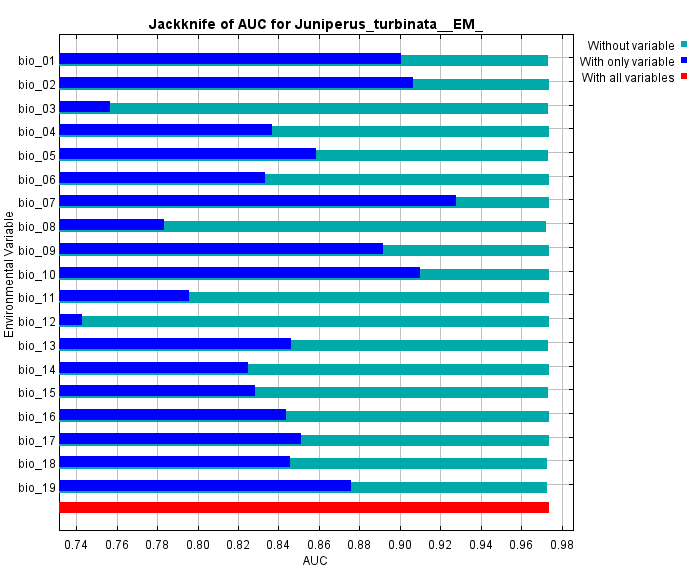


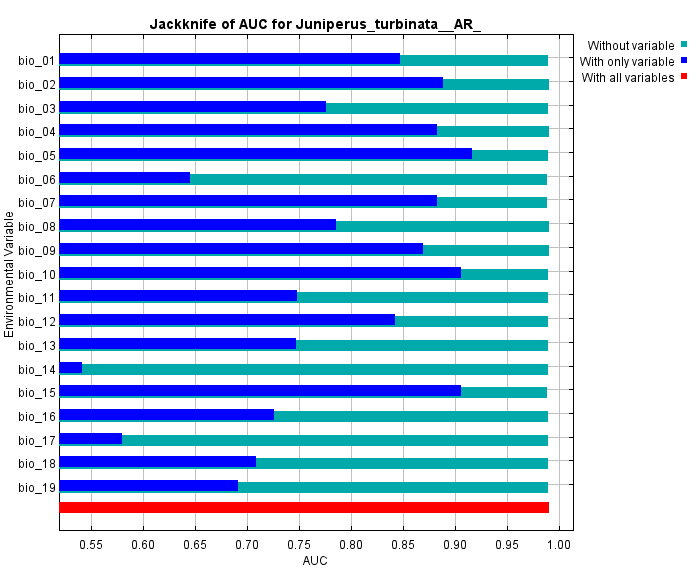
Figure S7. Jackknife test, using AUC (mean AUC=0.973) on test data of *Juniperus turbinata* (TUREM). The temperature annual range variable (bio_07), it is the variable that best generalizes, it is the most important variable to transfer past or future distributions under climate change.

Figure S8. Jackknife test, using AUC (mean AUC=0.990) on test data of *Juniperus turbinata* (TURAR). The max temperature of warmest month variable (bio_05), it is the variable that best generalizes, it is the most important variable to transfer past or future distributions under climate change. Mean temperature of warmest quarter (bio_10) and precipitation seasonality (bio_15) are also important to transfer distribution.
